# DLC1 promotes mechanotransductive feedback for YAP via RhoGAP-mediated focal adhesion turnover

**DOI:** 10.1242/jcs.261687

**Published:** 2024-04-30

**Authors:** Aukie Hooglugt, Miesje M. van der Stoel, Apeksha Shapeti, Beau F. Neep, Annett de Haan, Hans van Oosterwyck, Reinier A. Boon, Stephan Huveneers

**Affiliations:** ^1^ Amsterdam UMC, University of Amsterdam, Department of Medical Biochemistry, Amsterdam Cardiovascular Sciences, 1105AZ Amsterdam, the Netherlands; ^2^ Amsterdam UMC, VU University Medical Center, Department of Physiology, Amsterdam Cardiovascular Sciences, 1081HZ Amsterdam, the Netherlands; ^3^ KU Leuven, Department of Mechanical Engineering, Biomechanics section, 3001 Leuven, Belgium; ^4^ Amsterdam UMC, VU University Medical Center, Department of Pulmonary Medicine, Amsterdam Cardiovascular Sciences, 1081HZ Amsterdam, the Netherlands; ^5^ KU Leuven, Prometheus, Division of Skeletal Tissue Engineering, 3000 Leuven, Belgium; ^6^ German Center for Cardiovascular Research (DZHK), Partner Site Rhein-Main, 60590 Frankfurt am Main, Germany; ^7^ Goethe University, Institute of Cardiovascular Regeneration, 60590 Frankfurt am Main, Germany

**Keywords:** YAP/TAZ, Mechanotransduction, Focal adhesion, DLC1, Rho GTPase, Force, Endothelium, Migration, Angiogenesis, Integrin, Stiffness

## Abstract

Angiogenesis is a tightly controlled dynamic process demanding a delicate equilibrium between pro-angiogenic signals and factors that promote vascular stability. The spatiotemporal activation of the transcriptional co-factors YAP (herein referring to YAP1) and TAZ (also known WWTR1), collectively denoted YAP/TAZ, is crucial to allow for efficient collective endothelial migration in angiogenesis. The focal adhesion protein deleted-in-liver-cancer-1 (DLC1) was recently described as a transcriptional downstream target of YAP/TAZ in endothelial cells. In this study, we uncover a negative feedback loop between DLC1 expression and YAP activity during collective migration and sprouting angiogenesis. In particular, our study demonstrates that signaling via the RhoGAP domain of DLC1 reduces nuclear localization of YAP and its transcriptional activity. Moreover, the RhoGAP activity of DLC1 is essential for YAP-mediated cellular processes, including the regulation of focal adhesion turnover, traction forces, and sprouting angiogenesis. We show that DLC1 restricts intracellular cytoskeletal tension by inhibiting Rho signaling at the basal adhesion plane, consequently reducing nuclear YAP localization. Collectively, these findings underscore the significance of DLC1 expression levels and its function in mitigating intracellular tension as a pivotal mechanotransductive feedback mechanism that finely tunes YAP activity throughout the process of sprouting angiogenesis.

## Teaser

**Highlighted Article:** DLC1 inhibits basal Rho signaling to provide negative feedback for YAP activity during collective migration and sprouting angiogenesis.

## INTRODUCTION

Sprouting angiogenesis, the process through which new blood vessels are formed from pre-existing vessels, is essential for development and wound healing (Hillen and Griffioen, 2007; Potente et al., 2011). Disturbed angiogenesis contributes to the progression of cancer and cardiovascular disease (Fallah et al., 2019; Dudley and Griffioen, 2023). The collective migration and proliferation of vascular endothelial cells lie at the basis of sprouting angiogenesis, which is driven by the remodeling of their cell–cell and cell–extracellular matrix (ECM) adhesions (Eilken and Adams, 2010; Geudens and Gerhardt, 2011; Betz et al., 2016; Szymborska and Gerhardt, 2018). These multiprotein adhesion structures are intracellularly connected to the contractile actin cytoskeleton, providing a mechanical link between the cytoskeleton and endothelial microenvironment (Huveneers et al., 2015; Kechagia et al., 2019). Cellular adhesions sense the mechanical cues subjected to the endothelium (for example ECM stiffness, blood pressure and blood flow), and convert these cues into proportional intracellular adaptations. Such mechanotransduction responses safeguard proper endothelial cell proliferation, migration and sprouting during angiogenesis (Geudens and Gerhardt, 2011; Dorland and Huveneers, 2017).

Yes-associated protein (YAP; also known as YAP1) and its paralog transcriptional co-activator with PDZ-binding motif (TAZ; also known as WWTR1) (collectively YAP/TAZ) are transcriptional co-factors that are highly responsive to external and internal mechanical forces (Dupont et al., 2011). YAP/TAZ acts as a molecular switch and translocate to the nucleus upon their activation (Pocaterra et al., 2020). Nuclear YAP/TAZ interacts with transcription factors, in particular with the TEAD proteins, to drive the expression of pro-proliferative, pro-migratory and anti-apoptotic genes (Piccolo et al., 2014; Meng et al., 2016; Pocaterra et al., 2020). Activation of YAP/TAZ leads to remodeling of the actin cytoskeleton, focal adhesions and cell–cell junctions, all in support of endothelial cell migration and the angiogenic process (Nardone et al., 2017; Neto et al., 2018; Mason et al., 2019). Endothelial-specific conditional knockout of YAP/TAZ in mice leads to defects in vascular development, underscoring the critical role of YAP/TAZ in the vascular system (Choi et al., 2015; Kim et al., 2017; Wang et al., 2017; Neto et al., 2018). Conversely, induced expression of constitutively active YAP/TAZ mutants leads to vascular hypersprouting (Neto et al., 2018). These findings emphasize the necessity for finely tuned YAP/TAZ activity in the developing vasculature.

Physical forces within the vasculature, such as ECM stiffness, stretch, and shear stress, regulate YAP/TAZ signaling (Halder et al., 2012). In turn, YAP/TAZ activation levels are regulated by their own downstream targets through feedback loops in the signal transduction pathway (Hooglugt et al., 2020). It has been recently found that mechanical activation of YAP/TAZ limits cytoskeletal and focal adhesion maturation through a temporary transcriptional feedback loop. This cytoskeletal–transcriptional feedback prevents excessive focal adhesion maturation and enables persistent endothelial migration during angiogenic sprouting (Mason et al., 2023). We previously demonstrated that deleted-in-liver-cancer-1 (DLC1; also known as STARD12 or ARHGAP7) is a direct target of activated YAP/TAZ (van der Stoel et al., 2020). DLC1 contains a GAP domain, which inhibits Rho GTPase activity. Furthermore, DLC1 is recruited to focal adhesions, via its interaction with tensin, talin and focal adhesion kinase (FAK) proteins (Shi et al., 2012; Shih et al., 2015; Haining et al., 2018; van der Stoel et al., 2020). We, and others, have further shown that DLC1 is needed for focal adhesion disassembly, contact inhibition, collective cell migration and angiogenic sprouting (Shih et al., 2017; Sánchez-Martín et al., 2018; van der Stoel et al., 2020). Interestingly, DLC1 has also been identified as an upstream regulator of YAP activity to limit proliferation in confluent monolayers (Ritchey et al., 2019). Together, these findings suggest a potential role of DLC1 as a feedback effector for YAP/TAZ.

In the present study, we uncover a negative feedback loop between DLC1 expression and YAP activity during collective migration and sprouting angiogenesis. Our results reveal that DLC1, functioning as a YAP effector, in turn reduces YAP transcriptional activity and promotes angiogenic sprouting in a RhoGAP-dependent manner. DLC1 GAP activity prevents basal Rho signaling, thereby limiting cytoskeletal forces at the integrin–ECM adhesion interface. Collectively, these findings underscore the significance of DLC1 in modulating Rho signaling for mechanotransductive feedback to properly control YAP activity during sprouting angiogenesis.

## RESULTS

### DLC1 regulates nuclear–cytoplasmic translocation of YAP, and its transcriptional activity, in a RhoGAP-dependent manner

**Fig. 1.**
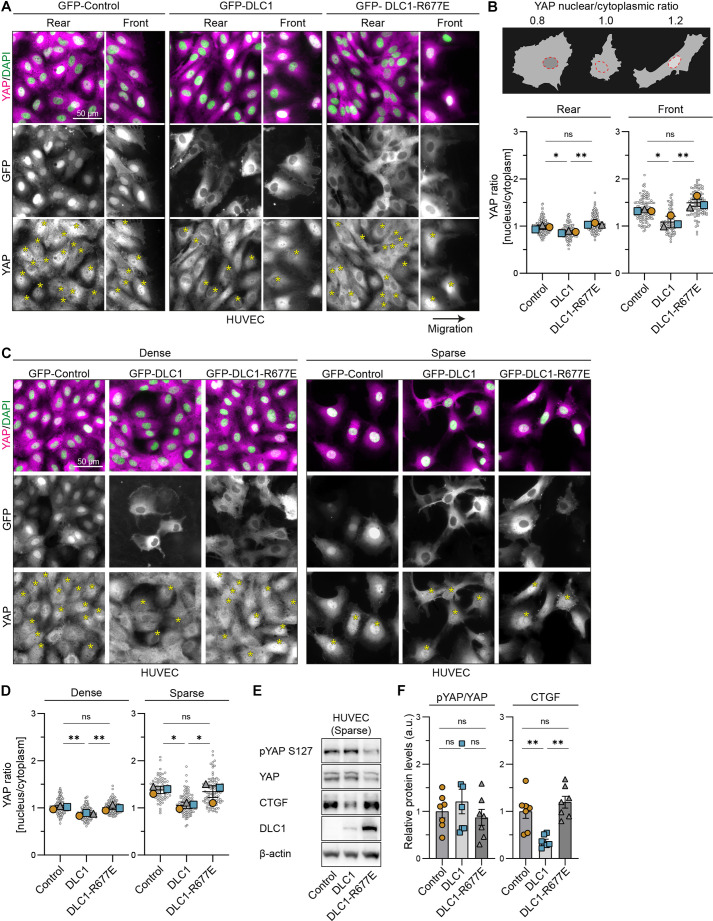
**DLC1 regulates YAP nuclear–cytoplasmic translocation and transcriptional activity through its RhoGAP function.** (A,C) Representative immunofluorescence images of HUVECs transduced with GFP (control), GFP–DLC1 or GFP–DLC1-R677E at the migration rear and front (A), and in dense or sparse (C) culture conditions. Stained for YAP (magenta) and DAPI (green). Yellow asterisks in the YAP grayscale images indicate GFP-positive cells. Cells in the scratch wound assay were fixed 4 h after scratch initiation. (B,D) Schematic representation of cells with different nuclear-to-cytoplasmic ratios of YAP intensity, reflecting low (0.8), intermediate (1.0) and high (1.2) levels of nuclear YAP (B, top). Graphs depicting the nuclear-to-cytoplasmic ratio of YAP intensity at the migration rear and front (B, bottom), and in dense or sparse (D) culture conditions. Gray circles represent the YAP ratio of individual cells. Total number of cells quantified (B) rear: control=206 cells, DLC1=159 cells, DLC1-R677E=181 cells; (B) front: control=131 cells, DLC1=89 cells, DLC1-R677E=108 cells; (D) dense: control=150 cells, DLC1=150 cells, DLC1-R677E=150 cells; (D) sparse: control=74 cells, DLC1=120 cells, DLC1-R677E=101 cells. The median YAP ratio of three independent experiments is presented and was tested by one-way ANOVA, Tukey's multiple comparisons test. (E) Representative western blot analysis of lysates from sparsely cultured HUVECs transduced with GFP (control), GFP–DLC1 or GFP–DLC1-R677E. Blotted for phospho-YAP S127 (pYAP), YAP, CTGF, DLC1 and β-actin (loading control). (F) Bar graphs show the ratio between pYAP S127 and YAP, and CTGF levels. a.u. arbitrary units. Data is from *n*=7 independent experiments; one-way ANOVA, Tukey's multiple comparisons test. All error bars are mean±s.e.m. ns, not significant; *P<0.05; **P<0.01.

To investigate whether DLC1 controls YAP, we overexpressed GFP (control) or GFP-tagged DLC1 (GFP–DLC1) in human umbilical vein endothelial cells (HUVECs). YAP localization was analyzed within migrating endothelial sheets during scratch wound assays, as well as in sparse and dense cell conditions. In control conditions YAP nuclear localization levels were highest at the migration front and in sparse densities (Fig. 1A–D), consistent with previous reports (Dupont et al., 2011; Mason et al., 2019). DLC1 overexpression significantly reduced the nuclear-to-cytoplasmic ratio of YAP in all investigated cellular conditions, which was most evident at the migration front and at sparse cell density (Fig. 1B,D). These results indicate that increased expression levels of DLC1 promote YAP nuclear-to-cytoplasmic translocation.

RhoA-mediated actomyosin contractility drives nuclear YAP/TAZ translocation and activation (Dupont et al., 2011). To investigate whether DLC1, in turn, might inactivate YAP through the regulation of Rho GTPases, we overexpressed a DLC1 variant in which one of the crucial arginine fingers in its RhoGAP domain is mutated (GFP–DLC1-R677E), rendering the GAP domain catalytically inactive (Zhong et al., 2009; Schimmel et al., 2018). Intriguingly, the expression of DLC1-R677E in HUVECs did not reduce YAP nuclear-to-cytoplasmic ratio in any of the aforementioned culture conditions (Fig. 1A–D).

A primary pathway regulating YAP/TAZ activity and cytoplasmic retention involves Hippo signaling, which leads to the phosphorylation of YAP/TAZ by LATS kinases. This inactivates YAP/TAZ via cytoplasmic sequestration or targeting for ubiquitin-mediated degradation (Piccolo et al., 2014; Ma et al., 2019). Next, we examined the impact of DLC1 or DLC1-R677E overexpression on YAP in lysates of sparsely cultured HUVECs, observing that it did not alter YAP phosphorylation at the LATS consensus site S127 (Fig. 1E,F). We detected a reduction in total YAP protein levels upon overexpression of DLC1-R677E, which suggests that there might be cellular compensation for the absence of a functional DLC1-mediated feedback to YAP. To assess the importance of DLC1 for transcriptional activity of YAP, we examined CTGF, a key YAP target gene. CTGF protein levels were significantly downregulated upon the overexpression of DLC1, but not by DLC1-R677E (Fig. 1E,F). These results show that the RhoGAP function of DLC1 inhibits YAP-mediated transcription through cytoplasmic retention of YAP, independent of YAP phosphorylation.

**Fig. 2.**
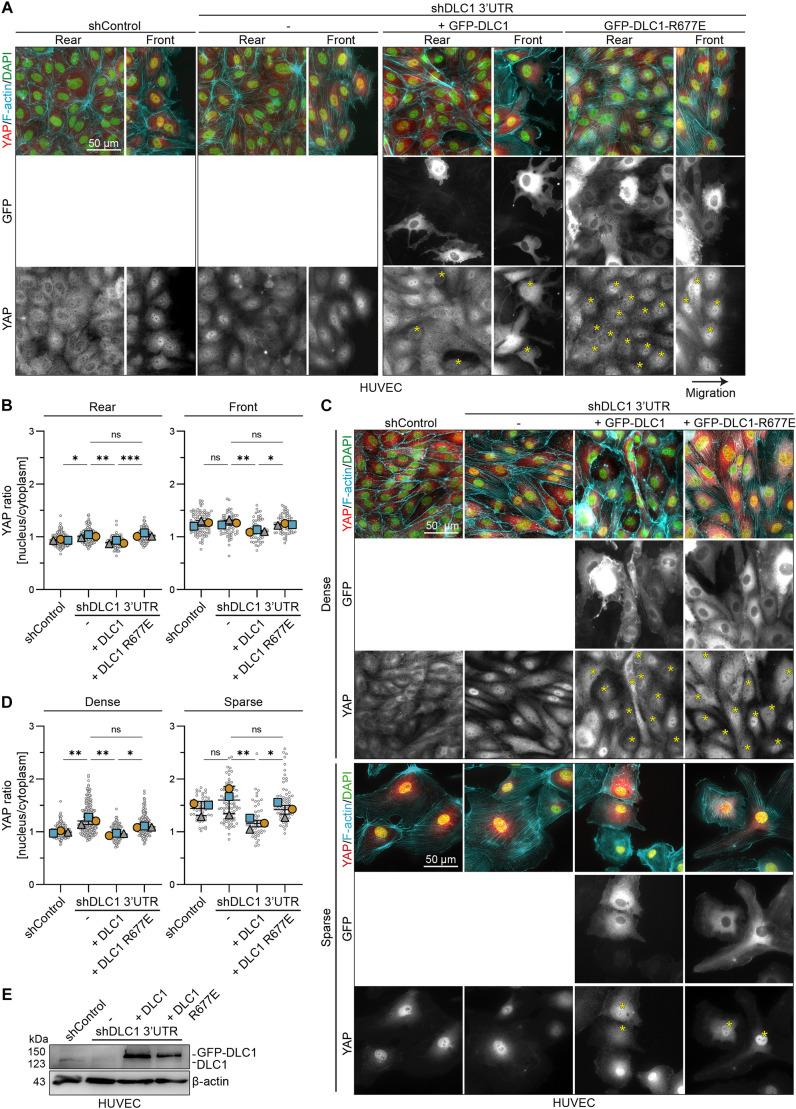
**DLC1, and its RhoGAP function, are needed to inhibit YAP translocation to the nucleus.** (A,C) Representative immunofluorescence images of HUVECs transduced with shControl, shDLC1 3′UTR and subsequently rescued with GFP–DLC1 or GFP–DLC1-R677E at the migration rear and front (A), and in dense or sparse (C) culture conditions. Stained for YAP (red), F-actin (cyan) and DAPI (green). Yellow asterisks in the YAP grayscale images indicate GFP-positive cells. Cells in the scratch wound assay were fixed 4 h after scratch initiation. (B,D) Graphs depicting the nuclear-to-cytoplasmic ratio of YAP intensity at the migration rear and front (B), and in dense or sparse (D) culture conditions. Gray circles represent the YAP ratio of individual cells. Total number of cells quantified (B) rear: control=265 cells, shDLC1=259 cells, shDLC1+DLC1=136 cells, shDLC1+DLC1-R677E=168 cells; (B) front: control=93 cells, shDLC1=70 cells, shDLC1+DLC1=61 cells, shDLC1+DLC1-R677E=76 cells; (D) dense: control=245 cells, shDLC1=236 cells, shDLC1+DLC1=129 cells, shDLC1+DLC1-R677E=196 cells; (D) sparse: control=60 cells, shDLC1=87 cells, shDLC1+DLC1=52 cells, shDLC1+DLC1-R677E=66 cells. The median YAP ratio of three independent experiments is presented and was tested by one-way ANOVA, Tukey's multiple comparisons test. (E) Representative western blot analysis from three repeats of lysates from HUVECs transduced with shControl or shDLC1 3′UTR and subsequently rescued with GFP-DLC1 or GFP–DLC1-R677E. Blotted for DLC1 and β-actin (loading control). All error bars are mean±s.e.m. ns, not significant; *P<0.05; **P<0.01; ***P<0.001.

To assess the significance of endogenous DLC1 on YAP localization, we depleted DLC1 using validated shRNAs targeting the 3′UTR of its mRNA (van der Stoel et al., 2020). DLC1 knockdowns led to an increase in nuclear YAP levels in the rear of migration endothelial monolayers (Fig. 2A,B). Furthermore, the depletion of DLC1 resulted in increased nuclear translocation of YAP in dense monolayers (Fig. 2C,D), consistent with previous literature (Ritchey et al., 2019). Knocking down endogenous DLC1 expression did not further increase the already high nuclear YAP levels of cells at sparse cell density or at the migration front (Fig. 2A–D). Restoring DLC1 protein levels by GFP–DLC1 expression drove YAP readily out of the nucleus in all conditions, whereas expressing the GFP–DLC1-R677E mutant did not affect the YAP nuclear-to-cytoplasmic ratio compared to that in DLC1 knockdown cells (Fig. 2A–E). Taken together, these results clearly demonstrate that DLC1, and its RhoGAP function, are needed for its negative feedback towards YAP activation.

### The RhoGAP function of DLC1 is essential for YAP-driven angiogenic sprouting

**Fig. 3.**
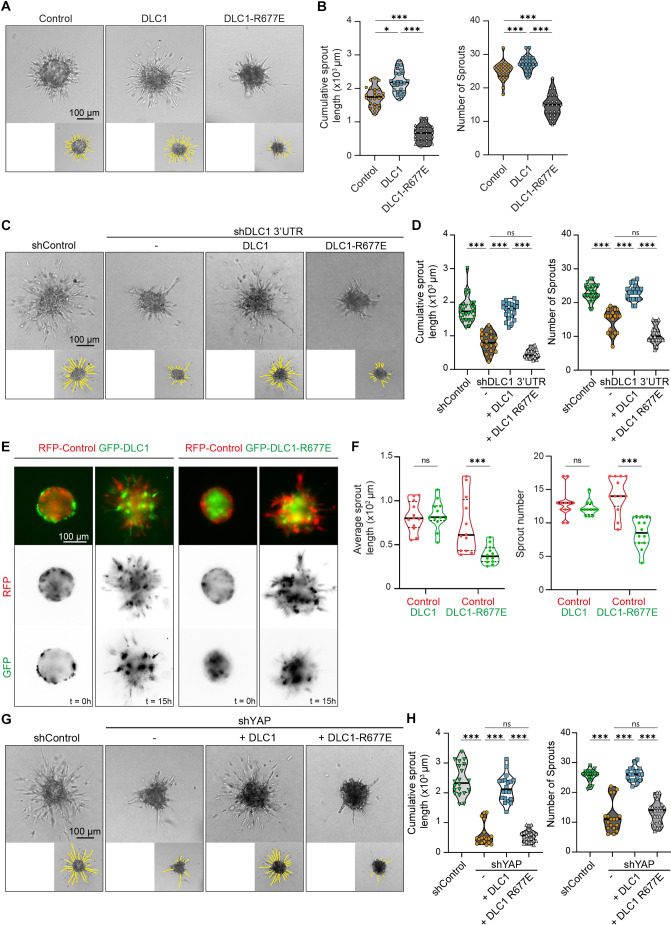
**The RhoGAP function of DLC1 is essential for YAP-driven angiogenic sprouting.** (A) Representative widefield images of spheroid-based sprouting assays of HUVECs transduced with GFP control, GFP–DLC1 or GFP–DLC1-R677E at 16 h after stimulation with VEGF. (B) Violin plots depict the cumulative sprout length and sprout number. Data is from *n*=3 independent experiments, control=25 spheroids, DLC1=25 spheroids, DLC1-R677E=24 spheroids; one-way ANOVA, Tukey's multiple comparisons test. (C) Representative widefield images of spheroid-based sprouting assay with HUVECs transduced with shControl, shDLC1 3′UTR, and subsequently rescued with GFP–DLC1 or GFP–DLC1-R677E at 16 h after stimulation with VEGF. (D) Violin plots depict the cumulative sprout length and sprout number. Data is from *n*=3 independent experiments, shControl=27 spheroids, shDLC1 3′UTR=19 spheroids, shDLC1+DLC1=21 spheroids, shDLC1+DLC1-R677E=19 spheroids; one-way ANOVA, Bonferroni's test. (E) Representative immunofluorescence images of competition sprouting assay with control (RFP) and GFP–DLC1, or control (RFP) and GFP–DLC1-R677E spheroids at the onset of VEGF stimulation and 15 h after VEGF stimulation. (F) Violin plots depicting average sprout length and sprout number of control (RFP), GFP–DLC1 or GFP–DLC1-R677E positive sprouts. Data is from *n*=3 independent experiments, DLC1 spheroids=12, DLC1-R677E spheroids=12; two-way ANOVA, Bonferroni's test. (G) Representative widefield images of spheroid-based sprouting assays of HUVECs transduced with shControl, shYAP and subsequently transduced with GFP–DLC1 or GFP–DLC1-R677E at 16 h after stimulation with VEGF. (H) Violin plots depict the cumulative sprout length and sprout number. Data is from *n*=3 independent experiments, shControl=20 spheroids, shYAP=18 spheroids, shYAP+DLC1=20 spheroids, shDLC1+DLC1-R677E=23 spheroids; one-way ANOVA, Bonferroni's test. In violin plots, dashed bars indicate the quartiles and solid bars the median.

Angiogenic sprouting is triggered by local inactivation of Rho signaling in endothelial cells (Abraham et al., 2009; Fischer et al., 2009; Guo et al., 2022). We have previously shown that DLC1 is essential for endothelial cell migration during angiogenic sprouting (van der Stoel et al., 2020). To investigate the role of the RhoGAP function of DLC1 in this process, we conducted VEGF-induced spheroid-based sprouting assays. Overexpression of DLC1 enhanced the angiogenic sprouting capacity of endothelial cells, whereas DLC1-R677E strongly attenuated sprouting (Fig. 3A,B). Moreover, ectopic expression of DLC1, but not DLC1-R677E, rescued the sprouting capacity of DLC1 knockdown cells (Fig. 3C,D). Collectively, these experiments show that DLC1 promotes angiogenic sprouting in a GAP-dependent manner.

To investigate whether the inability of DLC1-R677E overexpressing cells to sprout is due to their failure to acquire leader cell properties, we performed mosaic competition sprouting assays. Mosaic spheroids were generated in which half of the HUVECs were control (expressing RFP) and the other half expressed GFP–DLC1 or GFP–DLC1-R677E. At the onset of VEGF-induced sprouting, a difference in localization between distinct cells was already observed – in GFP–DLC1 and RFP mosaic spheroids, the GFP–DLC1-positive cells predominantly occupied the periphery, whereas in GFP–DLC1-R677E and RFP spheroids, the control cells were peripheral (Fig. 3E). After 15 h of VEGF induction, GFP- and RFP-positive cells contributed equally to sprout formation from GFP–DLC1 and RFP spheroids (Fig. 3F). By contrast, sprouts from GFP–DLC1-R677E and RFP spheroids mainly consisted of RFP-positive cells, and GFP–DLC1-R677E cells remained in the spheroid (Fig. 3F). These results demonstrate that the migratory capacity of DLC1-R677E-expressing cells is impaired during angiogenic sprouting, even in the presence of neighboring control leader cells.

Next, we investigated the importance of DLC1 RhoGAP activity for YAP-mediated angiogenic sprouting. shRNA-mediated knockdown of YAP in HUVECs significantly impairs angiogenic sprouting, which can be restored by ectopic expression of its downstream target DLC1 (Fig. 3G,H; van der Stoel et al., 2020). However, the expression of DLC1-R677E failed to restore the sprouting defects of YAP knockdown HUVECs (Fig. 3G,H). These findings demonstrate the importance of the RhoGAP function of DLC1 during its role as an effector of YAP in sprouting angiogenesis.

### DLC1 controls focal adhesion turnover and maturation in a RhoGAP-dependent manner

**Fig. 4.**
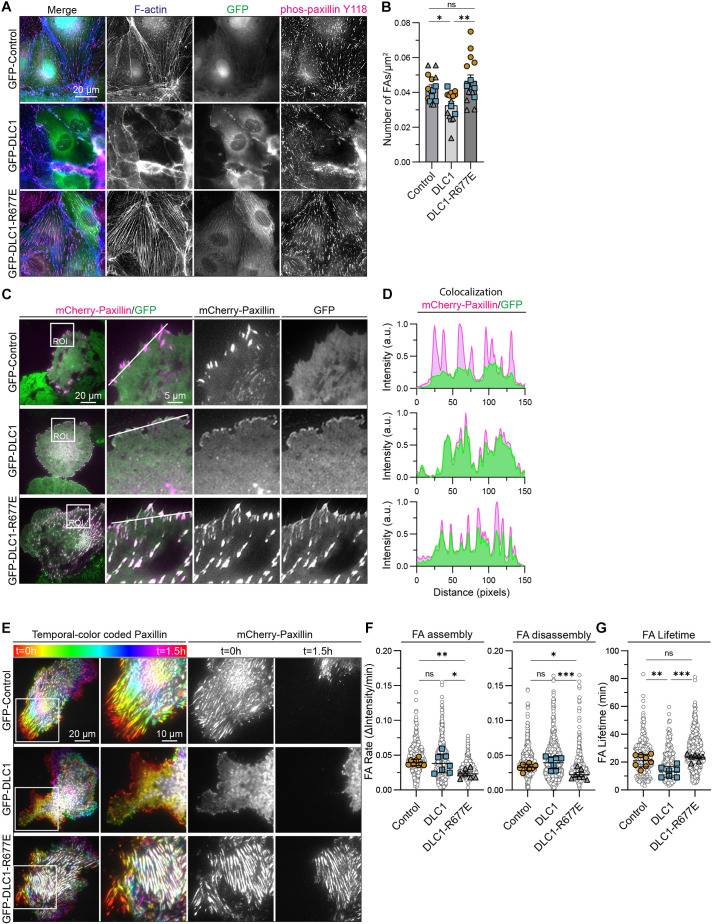
**DLC1 controls focal adhesion turnover and maturation in a RhoGAP-dependent manner.** (A) Representative immunofluorescence images of HUVECs expressing GFP (control), GFP–DLC1 or GFP–DLC1-R677E (green) and stained for F-actin (blue) and phosphorylated paxillin Y118 (magenta). (B) Bar graph shows the number of focal adhesions per cell, corrected for cell surface area. Focal adhesions were determined in 5 GFP-positive cells per condition for each independent experiment (*n*=3, represented by different symbols); one-way ANOVA, Tukey's multiple comparisons test. (C) Representative TIRF images from four repeats of live imaged HUVECs transduced with GFP (control), GFP–DLC1 or GFP–DLC1-R677E and mCherry–paxillin. See Movie 1 for the corresponding 3 h time-lapse recording. (D) Line graph analysis showing the colocalization of mCherry–paxillin with GFP–DLC1 and GFP–DLC1-R677E signal in live imaged HUVECs. a.u. arbitrary units. (E) Representative TIRF images of the mCherry–paxillin signal from live imaged HUVECs transduced with GFP (control), GFP–DLC1 or GFP–DLC1-R677E and mCherry–paxillin. Focal adhesion dynamics over 1.5 h are visualized by temporal color coding of mCherry–paxillin signal (1 color per frame, 1 frame/minute). Regions of interest (ROIs) highlight the contractile areas of the cells. Right panels display mCherry–paxillin signal in grayscale at timepoints *t*=0 h and *t*=1.5 h. (F,G) Graphs show the quantification of focal adhesion assembly and disassembly (F) and lifetime (G) based on TIRF timelapse imaging with HUVECs expressing GFP, GFP–DLC1 or GFP–DLC1-R677E and mCherry–paxillin. Gray circles represent each tracked focal adhesion, colored icons represent the median per cell. Data from *n*=4 independent experiments, the average number of focal adhesions tracked per cell: Control (329 assembly, 361 disassembly, 212 lifetime; 8 cells), GFP–DLC1 (234 assembly, 352 disassembly, 94 lifetime; 7 cells), GFP-DLC1-R677E (522 assembly, 577 disassembly, 323 lifetime; 7 cells). Time-lapse recordings were 3–4 h long and analyzed using the focal adhesion analysis server (Berginski and Gomez, 2013); one-way ANOVA, Tukey's multiple comparisons test. All error bars are mean±s.e.m. ns, not significant; *P<0.05; **P<0.01; ***P<0.001.

Because Rho GTPases control cytoskeletal and adhesion-mediated cellular remodeling, which are key upstream determinants of YAP/TAZ (Totaro et al., 2018), we hypothesized that DLC1-driven inactivation of YAP occurs through cellular remodeling. Indeed, overexpression of DLC1 in HUVECs resulted in reduced actin fibers and focal adhesions, which mostly localized at the cell periphery (Fig. 4A,B). Conversely, overexpression of DLC1-R677E promoted the formation of F-actin stress fibers and focal adhesions (Fig. 4A,B). The later phenotypic switch resembles the cellular changes that occur upon depletion of endogenous DLC1 in HUVECs (van der Stoel et al., 2020). A rescue of DLC1 expression in DLC1 knockdown HUVECs reduced the amounts of F-actin fibers and focal adhesions (Fig. S1). By contrast, expression of DLC1-R677E did not change stress fiber formation or focal adhesion levels in DLC1 knockdown cells (Fig. S1). These results indicate that DLC1-R677E acts as a dominant-negative protein.

As DLC1 binds to focal adhesion proteins, we next questioned whether the DLC1 GAP function acts on focal adhesion dynamics. To address this, we performed total internal reflection fluorescence (TIRF) live-cell microscopy in sparsely seeded (thus YAP activated) HUVECs expressing GFP control, GFP–DLC1 and GFP–DLC1-R677E together with the focal adhesion protein mCherry–paxillin. TIRF imaging showed that both GFP–DLC1 and GFP–DLC1-R677E were recruited to integrin-based focal adhesions (Fig. 4C,D; Movie 1). DLC1 overexpressing cells primarily form small and transient focal adhesions at the cell periphery, whereas DLC1-R677E overexpressing cells form large mature focal adhesions (Movie 1; Fig. 4E). Quantitative analysis using the established tracking software FAAS (Berginski and Gomez, 2013) demonstrated that the overexpression of DLC1 reduced the focal adhesion lifetime, but did not affect focal adhesion assembly or disassembly rates (Fig. 4F,G). By contrast, the overexpression of DLC1-R677E strongly reduced focal adhesion assembly and disassembly rates and induced many long-lived stationary focal adhesions in the cell body (Movie 1; Fig. 4E). Their lifetime could not be assessed through FAAS as they did not even turnover within the timeframe of the recordings; nevertheless the lifetime of other focal adhesions was slightly increased (Fig. 4G). Collectively, these results indicate that DLC1 RhoGAP activity promotes focal adhesion turnover and limits focal adhesion maturation.

### The RhoGAP function of DLC1 inhibits Rho signaling and traction forces

**Fig. 5.**
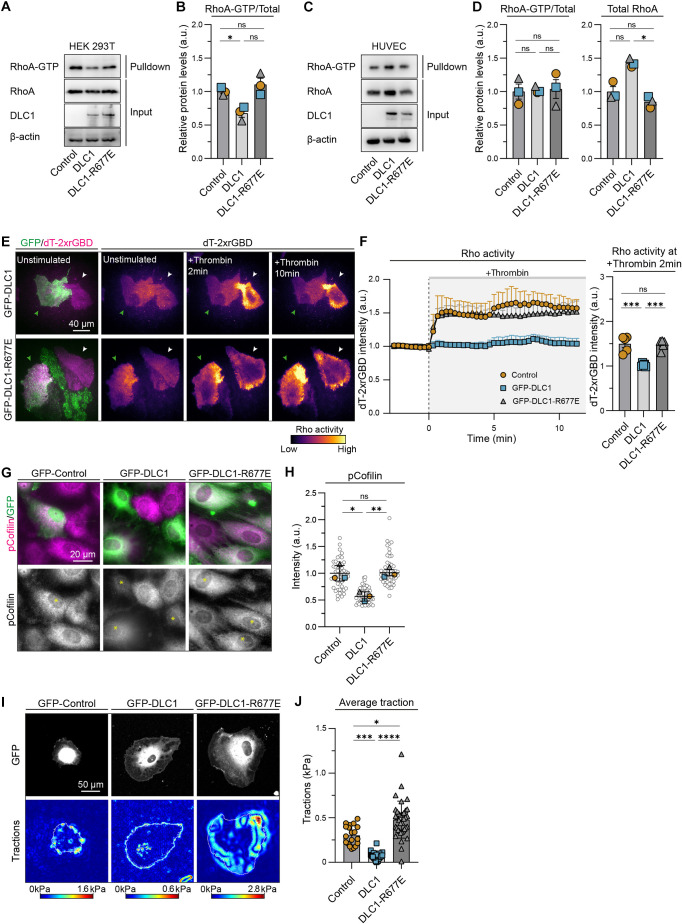
**The RhoGAP function of DLC1 inhibits basal Rho signaling and traction forces.** (A,C) Representative western blot analysis of RhoA-GTP levels in lysates from HEK 293T cells (A) and HUVECs (C) transduced with GFP (control), GFP–DLC1 or GFP–DLC1-R677E from rhotekin pull-downs. Blotted for RhoA, DLC1 and β-actin (loading control). (B,D) Bar graphs showing the ratio between RhoA-GTP and RhoA levels in HEK 293T cells (B) and HUVECs (D), and RhoA total protein levels in HUVECs (D). Data are from *n*=3 independent experiments; one-way ANOVA, Tukey's multiple comparisons test. (E) Representative TIRF images from live imaged HUVECs stimulated with thrombin and expressing the Rho biosensor dT-2xrGBD in control cells (white arrow), or in GFP–DLC1 or GFP–DLC1-R677E cells (green arrow). See corresponding Movie 2 for the 15 min time-lapse recording. (F) Normalized fluorescence intensity of the Rho biosensor dT-2xrGBD upon thrombin stimulation over time and at 2 min of stimulation. Data is from *n*=3 independent experiments, control=5 cells, DLC1=5 cells, DLC1-R677E=5 cells; one-way ANOVA, Tukey's multiple comparisons test. (G) Representative immunofluorescence images of HUVECs transduced with GFP (control), GFP–DLC1 or GFP–DLC1-R677E (green) and stained for phospho-cofilin S3 (pCofilin; magenta). Yellow asterisks in the grayscale images indicate GFP-positive cells. (H) The fluorescence intensity of pCofilin per cell. Gray circles represent the pCofilin intensity of individual cells. Data is from *n*=3 independent experiments, control=52 cells, DLC1=64 cells, DLC1-R677E=49 cells; one-way ANOVA, Tukey's multiple comparisons test. (I) Representative images of HUVECs transduced with GFP (control), GFP–DLC1 or GFP–DLC1-R677E and seeded on fluorescent bead-containing polyacrylamide gels (4 kPa) with corresponding heatmap of computed tractions. Arrows indicate direction of tractions. The cell edge is outlined in white. (J) Bar graph presents the average tractions per cell. Cells without any measureable tractions were not included in this quantification. Dots represent single cells: control=19 cells, DLC1=22 cells, DLC1-R677E=35 cells; Kruskal–Wallis, Dunn's multiple comparisons test. All error bars are mean±s.e.m. ns, not significant; *P<0.05; **P<0.01; ***P<0.001; ****P<0.0001.

DLC1 has been shown to inactivate RhoA in various mammalian cell types (Kim et al., 2007, 2013; Ko et al., 2010; Li et al., 2011; Cao et al., 2012; Du et al., 2012; Tripathi et al., 2012, 2014a,b; Shih et al., 2015; Wang et al., 2020). We confirmed the ability of DLC1 to reduce RhoA-GTP levels in human embryonic kidney cells (HEK 293T), whereas the RhoGAP-defective DLC1-R677E mutant did not inactivate RhoA, validating its loss-of-function (Fig. 5A,B). Surprisingly, the overexpression of DLC1 in HUVECs did not inhibit overall GTP-loading of RhoA (Fig. 5C,D). Instead, we consistently observed an increase in total RhoA protein levels upon DLC1 overexpression in HUVECs compared to what was seen with expression of DLC1-R677E (Fig. 5D), indicating possible compensatory effects within endothelial cells. Next, we investigated spatiotemporal Rho GTPase activity using a location-based Rho biosensor. This biosensor is composed of two rhotekin G protein-binding domains fused to dimericTomato (dT-2xrGBD) and reports the localization of endogenously active GTP-bound Rho GTPases (Mahlandt et al., 2021). We examined the effect of DLC1 on the relocation of the biosensor to the basal plasma membrane by TIRF microscopy. The endothelial cells were stimulated with thrombin, which rapidly resulted in sustained Rho activation (∼50% increase in intensity) in control cells (Movie 2; Fig. 5E,F). Strikingly, overexpression of DLC1, but not DLC1-R677E, prevented the thrombin-induced Rho activation in the basal plane of endothelial cells (Movie 2; Fig. 5E,F). Biochemical rhotekin pulldowns on corresponding cell lysates showed that thrombin efficiently promotes RhoA GTP-loading in control, DLC1 or DLC1-R677E conditions (Fig. S2A,B). Together, the data indicate that DLC1 does not inhibit global RhoA signaling, but limits the local activation of Rho GTPases at the basal plasma membrane.

To assess whether the inhibitory role of DLC1 on Rho signaling requires other protein regions in addition to its GAP domain, we generated a truncated GAP domain mutant and its catalytic inactive R677E variant. Upon their expression, we observed that these GAP domain variants are cytoplasmic and did not localize at the focal adhesions (Fig. S3A,B). Moreover, both GAP variants were enriched in the nucleus, whereas the full-length DLC1 protein counterparts were not, indicating that the other regions within the DLC1 protein contribute to its subcellular localization. Expression of the GAP domain of DLC1 reduced F-actin amounts and the formation of focal adhesions (Fig. S3B,C). Moreover, the GAP domain strongly induced membrane ruffles containing small punctate focal contacts at the cell periphery (Fig. S3C), indicating that expression of only the GAP domain had even stronger effects than expression of full-length DLC1, possibly due to the absence of its auto-inhibitory domains. In addition, the GAP domain inhibited YAP-mediated CTGF expression (Fig. S3D,E) and basal Rho activation by thrombin (Movie 3; Fig. S3F,G). Conversely, expression of the catalytically inactive GAP-R677E domain promoted formation of focal adhesions and stress fibers, and did not inhibit thrombin-induced basal Rho activation. These results show that the presence of DLC1 GAP activity in the cytoplasm is sufficient for the reported endothelial feedback responses.

Key effectors of RhoA are the Rho-associated coiled-coil containing kinases (ROCK1 and ROCK2, referred to here collectively as ROCK). Downstream targets of ROCK are the phosphorylation of cofilin (herein referring to both cofilin-1 and cofilin-2) and myosin light chain II (MLC2; also known as MYL9). Immunofluorescence analysis showed that overexpression of DLC1, but not DLC1-R677E, reduced cofilin phosphorylation (Fig. 5G,H). Because dephosphorylated cofilin depolymerizes actin filaments, this corresponds with the notion that DLC1 overexpressing cells lack F-actin stress fibers. We did not observe downregulation of MLC2 phosphorylation in DLC1 or DLC1-R677E overexpressing cells (Fig. S4A–C), suggesting that DLC1 does not directly control MLC2 activation. Taken together, these findings point to an inhibitory role for the GAP function of DLC1 on Rho-mediated phosphorylation of cofilin.

Rho–ROCK-mediated cytoskeletal remodeling governs intracellular tension, which in turn promotes YAP nuclear translocation (Dupont et al., 2011; Totaro et al., 2018; Li et al., 2023). Intracellular tension is transduced through focal adhesions to the extracellular matrix. To investigate the role of DLC1 on intracellular tension, we conducted traction force microscopy on 4 kPa polyacrylamide gels. DLC1 overexpression significantly reduced cell tractions (Fig. 5I,J). Intriguingly, many DLC1 overexpressing cells did not exert any traction at all, indicating that DLC1 has a prominent effect on traction forces. Conversely, DLC1-R677E overexpressing cells exerted higher traction forces compared to control cells (Fig. 5I,J). These results show that DLC1 RhoGAP activity strongly suppresses intracellular tension.

### DLC1 RhoGAP activity provides cytoskeletal-mediated feedback for YAP

**Fig. 6.**
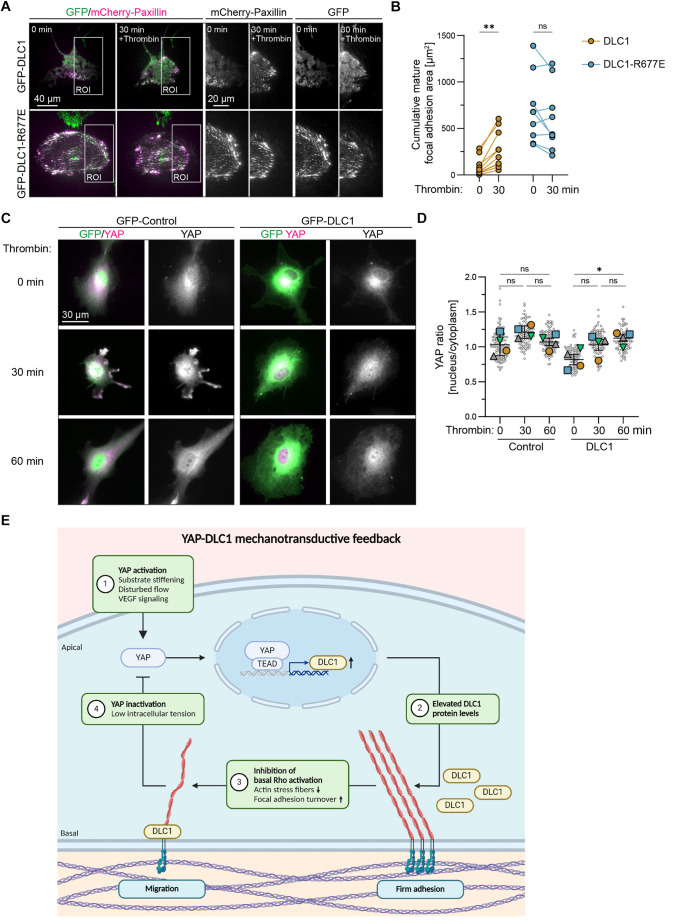
**DLC1 RhoGAP activity provides cytoskeletal force-mediated feedback for YAP.** (A) Representative TIRF images from live imaged HUVECs transduced with GFP (control), GFP–DLC1 or GFP–DLC1-R677E and mCherry–paxillin stimulated with thrombin. See corresponding Movie 4 for the 45 min time-lapse recording. (B) Graph shows the cumulative mature focal adhesion area per cell at 0 and 30 min of thrombin stimulation. Focal adhesions were considered mature when larger than 1 µm^2^. Data is from *n*=3 independent experiments, DLC1=10 cells, DLC1-R677E=9 cells; two-way ANOVA, Šídák's multiple comparisons test. (C) Representative immunofluorescence images of HUVECs transduced with GFP (control) or GFP–DLC1, cultured in sparse conditions. Cells were serum starved and fixed after 0 min, 30 min or 60 min of thrombin stimulation and stained for YAP (magenta). Of note, serum-starvation reduced basal nuclear YAP localization compared to cells in normal medium as shown in Fig. 1C. (D) Graphs depicting the nuclear-to-cytoplasmic ratio of YAP intensity in thrombin-stimulated control or DLC1-expressing cells. Gray circles represent the YAP ratio of individual cells. Total number of cells quantified: control 0 min=109 cells, 30 min=91 cells, 60 min=106 cells; DLC1 0 min=87 cells, 30 min=97 cells, 60 min=87 cells. The mean±s.e.m. YAP ratio of *n*=4 independent experiments is presented and tested by two-way ANOVA, Dunnett's multiple comparisons test. ns, not significant; *P<0.05; **P<0.01. (E) Graphical summary of the RhoGAP-mediated feedback loop of DLC1 on YAP activity. Created with BioRender.com.

Next, we questioned whether the DLC1-mediated suppression of intracellular tension functions as a continuously active negative feedback signal. Stimulating endothelial cells with thrombin leads to Rho-mediated actomyosin contractility and focal adhesion maturation (Amerongen et al., 2000; Huveneers et al., 2012; Botros et al., 2020). We examined thrombin-evoked focal adhesion remodeling by performing TIRF microscopy of mCherry–paxillin. Interestingly, upon addition of thrombin to DLC1 overexpressing cells, an immediate increase in focal adhesion number and size was observed (Movie 4; Fig. 6A,B), indicating a restoration of cellular adhesive capacity. Of note, thrombin induced normal remodeling of focal adhesions and cellular contraction in DLC1-R677E cells. These results show that negative feedback signaling from DLC1 towards intracellular tension can be bypassed by generating overall Rho-mediated contractility.

Next, we investigated the effect of thrombin on YAP localization in HUVECs overexpressing DLC1. Intriguingly, we observed a significant increase in nuclear YAP levels at 60 min after thrombin stimulation in sparsely cultured cells (Fig. 6C,D). This indicates that thrombin-induced Rho-mediated contractility restores nuclear YAP translocation even in the presence of high DLC1 levels. Together, these experiments demonstrate that the DLC1-driven mechanotransductive negative feedback loop actively suppresses YAP nuclear translocation.

## DISCUSSION

The mechanotransduction players YAP/TAZ have emerged as key transcriptional regulators of endothelial cell proliferation, migration and sprouting angiogenesis. Recent studies have revealed that various YAP/TAZ effectors fine-tune the activity of YAP/TAZ through feedback mechanisms, thereby facilitating collective endothelial migration and angiogenesis (Mason et al., 2019; Park et al., 2019; Hooglugt et al., 2020; Moon et al., 2020; van der Stoel et al., 2020). In this study, we identified a novel feedback loop between YAP and its target gene DLC1. Specifically, we demonstrated that signaling through the RhoGAP function of DLC1 leads to a reduction in the nuclear localization of YAP and its transcriptional activity (Fig. 6E). Furthermore, we demonstrated the importance of the RhoGAP function of DLC1 during its role as an effector of YAP in sprouting angiogenesis, thus establishing RhoGAP activity as a crucial negative feedback event to modulate YAP-mediated processes in sprouting angiogenesis.

### DLC1-mediated negative feedback to YAP/TAZ promotes sprouting angiogenesis

Angiogenesis is a tightly regulated process, wherein endothelial cells undergo an initial transition into a pro-angiogenic state. However, once vessel anastomosis occurs and the vessel lumen opens, the angiogenic activity of endothelial cells ceases, leading to stabilization of the vasculature (Potente et al., 2011; Betz et al., 2016). To achieve such tight regulation, endothelial cells require feedback systems that prevent, for instance, aberrant angiogenic hypersprouting. Transcriptional feedback loops, in which YAP/TAZ activation levels are regulated by their downstream targets, are likely important to sustain efficient endothelial migration during sprouting angiogenesis. Several YAP/TAZ effectors (specifically CTGF, CYR61 and NUAK2) have been described to control YAP/TAZ function by providing feedback in angiogenesis. CTGF promotes YAP expression levels and sprouting angiogenesis (Moon et al., 2020). CYR61 (also known as CCN1) inhibits YAP during physiological and pathological angiogenesis through remodeling of the actin cytoskeleton (Lee et al., 2019). Conversely, another study found that CYR61 promotes YAP/TAZ activity in tip cells via VEGFR2 signaling (Park et al., 2019). Another YAP/TAZ positive feedback loop occurs through SNF-like kinase 2 (NUAK2), a myosin light chain phosphatase regulator (Mason et al., 2019). NUAK2 promotes focal adhesion maturation and myosin II-mediated cytoskeletal contractility, which in turn further activates YAP/TAZ. YAP/TAZ-mediated changes in expression of DLC1, enables a balanced control over adhesion and cytoskeletal contractility for directed cell migration (van der Stoel et al., 2020). We now show that the increasing levels of YAP/TAZ–TEAD-mediated DLC1 expression will lead to sequestration of YAP in the cytoplasm and inhibition of YAP activity, overall contributing to efficient endothelial collective behavior. A primary determinant for YAP/TAZ activation is the cell shape, which in turn is controlled by the cytoskeletal structure and intracellular tension (Totaro et al., 2018). Although there are a few paralog-specific regulatory mechanisms described in literature, cell shape and cytoskeletal integrity is a common regulatory mechanism for both YAP and TAZ (Totaro et al., 2018; Reggiani et al., 2021). In this study, we focused on the interplay between YAP and DLC1. Given that DLC1 expression levels are also regulated by TAZ (van der Stoel et al., 2020), we suspect that DLC1 might also provide feedback to TAZ. Future research that is aimed at the unraveling of the spatiotemporal interplay of YAP/TAZ, DLC1 and other effectors would provide valuable insights into how feedback mechanisms work during vascular development and are perturbed in pathological vasculature.

The transcriptional activation of YAP is known to be controlled by a variety of mechanical and biochemical signals. In our study, we employed immunofluorescence and traction force microscopy techniques to elucidate the molecular mechanisms of DLC1-mediated feedback to YAP. These experiments revealed that DLC1 overexpression led to the development of immature cytoskeletal and focal adhesion structures, accompanied by a reduction in cellular tractions and intracellular tension. Given that these effects were dependent on the presence of a functional RhoGAP domain, we further explored targets downstream of Rho-ROCK signaling and identified cofilin proteins as being associated with the cytoskeleton-compromised phenotype of DLC1 overexpressing cells. Taken together, these findings demonstrate the ability of DLC1 to control endothelial cell shape by inhibition of Rho-cofilin signaling, which in turn leads to the attenuation of YAP activity. Inhibition of Rho GTPase signaling by Y-27632, a well-known inhibitor of ROCK, was previously shown to increase endothelial-mediated sprouting in 3D (Guo et al., 2022). The increase in migration capacity was attributed to the formation of short-lived, high turnover, focal adhesions. This corresponds with our finding that the formation of very static and strong focal adhesions, such as in DLC1-R677E-expressing cells or DLC1-depleted cells, negatively affects cell migration and sprouting. This is reminiscent of related 3D migratory processes, for instance during metastasis, when tumor cells change motility modes and higher migration velocity is marked by a reduction in focal adhesions and actin bundles (Chikina and Alexandrova, 2014).

### DLC1 interactions at focal adhesions controls its RhoGAP activity

The regulation of DLC1 RhoGAP activity involves various mechanisms. Intramolecular interactions between the RhoGAP domain and the SAM domain keeps DLC1 in an auto-inhibited state (Kim et al., 2007; Tripathi et al., 2014a). CDK5-mediated phosphorylation relieves the auto-inhibited conformation of DLC1 and promotes its focal adhesion recruitment and GAP activity (Tripathi et al., 2014a). At focal adhesions, various tensin isoforms have been found to interact with the SAM domain of DLC1, thereby controlling the auto-inhibited state of DLC1. Of note, there is no clear consensus yet whether tensin binding has a stimulatory, or potential inhibitory, effect on DLC1 RhoGAP activity (Qian et al., 2007; Chan et al., 2009; Cao et al., 2012; Shih et al., 2015; Joshi et al., 2020). Also talin- or FAK-binding-deficient DLC1 mutants retain their ability to inhibit Rho-GTP levels (Li et al., 2011). By expressing the truncated GAP domain of DLC1, we now confirm that focal adhesion recruitment is not needed per se to mediate basal Rho inhibition. Furthermore, DLC1 is involved in the crosstalk between Ras and Rho pathways at focal adhesions, where p120RasGAP (also known as RASA1) inhibits DLC1 through direct binding of its RhoGAP domain (Yang et al., 2009; Chau et al., 2022). The coordination of these various interactions in regulating DLC1 RhoGAP activity at focal adhesions remains poorly understood. We found that the dominant-negative DLC1-R677E reduced focal adhesion assembly, whereas we previously did not detect this in DLC1-depleted endothelial cells (van der Stoel et al., 2020). Potentially, the dominant-negative effect of DLC1-R667E expression, through its recruitment to focal adhesions, affects focal adhesion assembly more profoundly than in the absence of endogenous DLC1.

DLC1 has GAP activity towards the GTPases RhoA, RhoB, RhoC and, to a lesser extent, to Cdc42, Rac1 and TC10 (RhoQ) (Wong et al., 2003; Healy et al., 2008; Amin et al., 2016). A recent study has revealed that the dissociation of DLC1 from focal adhesions promotes local bursts of RhoA activity in response to mechanical stimuli (Heydasch et al., 2023). Using the same Rho biosensor, we find that DLC1 limits thrombin-induced Rho activation in the basal plasma membrane, whereas overall Rho GTP-loading still occurs and is sufficient to rescue YAP translocation. Interestingly, DLC1 has been reported to be recruited to mature focal adhesions through a force-regulated interaction with talin proteins to inhibit focal adhesion growth (Haining et al., 2018). We postulate that the force-controlled binding of DLC1 to talins triggers inhibition of Rho GTPases, which in turn modulate F-actin and focal adhesion maturation, thereby influencing intracellular tension, YAP activity and ultimately angiogenic sprouting.

### Concluding remarks

Hyperactivation of YAP/TAZ is observed in many forms of cancer, which promotes a pro-angiogenic, pro-inflammatory and hypoxic tumor microenvironment (Hooglugt et al., 2020). Angiosarcoma, an endothelial-derived form of cancer, is characterized by perturbed angiogenesis and the loss of DLC1 (Azad et al., 2019). DLC1-deficient angiosarcoma cells in tumors display increased nuclear YAP levels (Ritchey et al., 2019). Our current study demonstrates that DLC1 exerts its inhibitory effect on YAP signaling by regulating intracellular tension through its GAP function. Jointly, these observations provide a rationale for the development of targeted therapies that harness this protective function of DLC1 on intracellular tension in cancer.

## MATERIALS AND METHODS

### Antibodies and reagents

Purified mouse-anti human DLC1 [clone 3, cat. no. 612021, diluted 1:1000 for western blotting (WB)], was obtained from BD Biosciences. Rabbit anti-phospho-paxillin (Y118) antibody (cat. no. 69363S, diluted 1:400 for immunofluorescence, 1:1000 for WB), rabbit anti-phospho-MLC2 S19 antibody (cat. no. 3671S, 1:1000 for WB), rabbit anti-MLC2 antibody (cat. no. 8505S, 1:1000 for WB), rabbit anti-phospho-cofilin S3 antibody (cat. no. 3313S, 1:100 for WB), and rabbit anti-RhoA antibody (cat. no. 2117S, 1:1000 for WB) were purchased from Cell Signaling Technology. Mouse monoclonal anti-YAP1 antibody (63.7, cat. no. sc-101199, diluted 1:100 for immunofluorescence) was obtained from Santa Cruz Biotechnology. As a loading control, we used rabbit polyclonal anti-β-actin (Cell Signaling, Cat. no. 4967S, diluted 1:1000 for WB). To visualize F-actin we used PromoFluor 415-Phalloidin (cat. no. PK-PF415-7-01, diluted 1:200 for immunofluorescence) or PromoFluor 488-Phalloidin (cat. no. PK-PF488P-7-01, diluted 1:200 for immunofluorescence) from PromoKine. To visualize the nucleus, DAPI (Invitrogen, diluted 1:1000 for immunofluorescence) was used. Secondary antibodies coupled to Alexa Fluor 488 or 594 were from Invitrogen (diluted 1:250 for immunofluorescence). Secondary antibodies coupled to horseradish peroxidase (HRP) were obtained from Bio-Rad (diluted 1:1000 for WB).

### Cell culture

Pooled primary human umbilical vein endothelial cells (HUVECs) from different donors (Lonza) were cultured in Endothelial Cell Growth Medium 2 culture medium supplemented with the Growth Medium 2 Supplement Pack (PromoCell) on gelatin-coated tissue flasks. HEK293T cells (ATCC) were cultured in Dulbecco's modified Eagle's medium (DMEM) with L-glutamine and supplemented with 10% FCS and 1% penicillin-streptomycin. Cells were recently authenticated and tested for contamination.

### DNA plasmids and lentivirus production

shControl (shC002) lentiviral constructs and the shRNA in the lentiviral pLKO.1 backbone targeting YAP1 (TRCN107265) were from the Sigma-Aldrich mission library. A modified version of a pLKO.1 plasmid targeting DLC1 was generated based on the 5′-GGAGTGTAGGAATTGACTATA-3′ sequence to express shRNA that targeted the 3′-UTR of human DLC1 mRNA. Full-length human DLC1 fused at its N-terminus to a GFP tag was amplified by PCR from a pEGFP-C1-DLC1 vector and cloned into a self-inactivating lentiviral pLV-CMV-ires-puro vector between the SnaBI and XbaI restriction sites as described before (van der Stoel et al., 2020). The arginine to glutamic acid mutation at amino acid position 677 was generated by site-directed mutagenesis to generate the GFP-DLC1-R677E lentiviral construct as described before (Schimmel et al., 2018). The truncated GAP domain mutant and its catalytic inactive R677E variant were amplified by PCR from amino acid position 610 to 877 and cloned into a self-inactivating lentiviral pLV-CMV-MCS-eGFP-ires-puro vector at the XhoI site. The plasmids can be obtained from the corresponding author upon request.

### Immunofluorescence staining

For standard immunofluorescence staining, cells were cultured in sparse (10–20% confluency) or dense (100% confluency) conditions on glass coverslips coated with 5 µg ml^−1^ fibronectin. Cells were fixed with 4% PFA in PBS^++^ (PBS supplemented with 1 mM calcium chloride and 0.5 mM magnesium chloride) for 10 min at room temperature. Subsequently, fixed samples were permeabilized for 5 min with 0.5% Triton X-100 in PBS and blocked for 10 min with 2% BSA in PBS. Primary and secondary antibodies were diluted in 0.5% BSA in PBS and incubated for 45 min. After antibody incubation, cells were washed three times with 0.5% BSA in PBS. Coverslips were mounted in Mowiol4-88 with DABCO solution.

### Sprouting angiogenesis assay

For sprouting angiogenesis assays, HUVECs were seeded in EGM-2 medium containing 0.1% methylcellulose (4000 cP, Sigma-Aldrich). Spheroids were formed from 750 cells per 100 µl methylcellulose medium, seeded in a U-bottom well plate (CELLSTAR, cat. no. 650185) and incubated overnight. Spheroids were collected, and resuspended in 1.7 mg/ml collagen type I rat tail mixture (IBIDI) and plated in a glass bottom 96-well plate (Greiner, cat. no. 655892). Spheroids were stimulated with 50 ng ml^−1^ VEGF (human VEGF 165; Peprotech, cat. no. 100-20-50) in EGM2 and incubated overnight as previously described (Korff and Augustin, 1999). Sprouting was analyzed by taking pictures 16 h after VEGF stimulation using the EVOS M7000 imaging system and a 10× objective. Images were enhanced for display using an unsharp mask filter and adjusted for brightness and contrast. Sprouts were analyzed using the NeuronJ plugin in ImageJ. Spheroids in the mosaic sprouting experiments were formed by mixing of GFP-positive and RFP-positive cell populations. Mosaic sprouting was analyzed by assessing the sprout outgrowth in the GFP and RFP channel using the NeuronJ plugin.

### Fluorescence microscopy

For widefield imaging of fixed samples, a NIKON eclipse TI equipped with 60×1.49NA Apo TIRF (oil) objective, a lumencor SOLA SEII light source and standard DAPI, CFP, GFP, mCherry and Cy5 filter cubes were used. For fluorescence microscopy of mosaic sprouting angiogenesis, the inverted NIKON eclipse TI with a 20× long working distance (dry) objective was used. TIRF microscopy was performed using a NIKON eclipse TI equipped with a 60×1.49NA Apo TIRF (oil) objective, perfect focus system, argon laser 488 nm (Melles Griot), orange diode solid state laser 594 nm (Excelsior, Spectra-physics), dual band 488/594 nm TIRF filter cube (Chroma TRF59905 ET), 440/514/561 TIRF filter cube (Chroma zt440/514/561rpc) and an Andor Zyla 4.2 plus sCMOS camera. An Okolab cage incubator and humidified CO_2_ gas chamber set to 37°C and 5% CO_2_ were used during imaging. A 1-min interval was used during image acquisition for 3 h. Focal adhesion dynamics were analyzed using the Focal Adhesion Analysis Server (Berginski and Gomez, 2013). The raw data was uploaded using a minimal adhesion size of 4 pixels and a phase length of 5 min. To capture thrombin-induced Rho GTPase activation, images were acquired every 10 s for a total recording of 15 min (3 min before and 12 min after thrombin stimulus). The increase in intensity of the dT-2xrGBD sensor upon thrombin stimulation was normalized to the respective baseline intensity of the cell prior to stimulation.

### Traction force microscopy

Traction force microscopy experiments were performed on bead-bound polyacrylamide hydrogels (4 kPa gels, 0.2 μm beads) purchased from Matrigen (cat. no. SW24G-EC-4-ST0.2R) and imaged with a Leica TCS SP8 confocal laser scanning microscope, with 40× oil (1.30 NA) and 63× oil (1.40 NA) objectives using 470–670-nm white light lasers. An in-house MATLAB code was used for image analysis (available upon request). Bead images were filtered to suppress noise using a Gaussian filter followed by binarization of the cell image using thresholding. Any possible stage shifts during imaging were corrected by means of a phase correlation-based global rigid registration. A previously described free form deformation-based (FFD) registration method was employed to calculate cell-induced displacements of the beads by using a grid of control points that define a B-spline non-rigid transformation (Jorge-Peñas et al., 2015). The open-source image registration toolbox Elastix (https://simpleelastix.github.io/) was used with advanced normalized correlation as the registration metric and the quasi-Newton method for optimization. A grid size of 15 pixels was used, which guaranteed sufficient beads between control points and a 99% registration metric for most cases. A Tikhonov-regularized Fourier transform traction cytometry algorithm was applied with the assumption of a homogeneous, isotropic and linear elastic half space. Given that the noise observed in the experimental beads data was minimal, a fixed minimum amount of regularization of *e*^−6^ was applied. Finally, cell tractions were recovered using the regularized inversion of the elasticity problem in the Fourier domain. Traction footprints were then obtained by applying an Otsu thresholding algorithm to the recovered tractions (Izquierdo-Álvarez et al., 2019). The average tractions (||tavg||) shown in Fig. 5I were calculated within these footprints using the following formula:

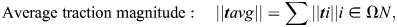


where Ω is the traction footprint region, *N* is the number of pixels in Ω, *ti* is the traction vector at spatial point *i*.

### Western blot analysis

Cells were lysed in ice-cold RIPA buffer (150 mM sodium chloride, 50 mM 1 M Tris-HCl pH 8, 1 mM EDTA, 1% NP40, 0.1% SDS and 0.5% sodium deoxycholate) supplemented with protease inhibitor cocktail (1× PIC, Sigma-Aldrich) and phosphatase inhibitor (0.01 M NaF). For the rhotekin pulldown assay, rhotekin–RBD beads were purchased from Cytoskeleton (cat. no. RT02) and cell lysis and pulldown was performed as indicated by the manufacturer. Protein concentration was measured using the detergent compatible (DC) protein assay (BioRad, #5000112), and protein solutions were diluted accordingly in RIPA and reduced sample buffer with 4% β-mecaptoethanol. Samples were denatured at 95°C for 5–10 min and subsequently loaded on a 10% SDS-Page gel or precast 4–12% gradient gels (Thermo Fisher Scientific, cat. no. NW04125BOX). Gel electrophoresis was performed in SDS-PAGE running buffer (25 mM Tris-HCl pH 8.3, 192 mM glycine and 0.1% SDS) or MOPS buffer (Thermo Fisher Scientific, cat. no. NP0001). Proteins were transferred to ethanol-activated PVDF membranes using wet transfer in Towbin blot buffer [25 mM Tris-HCl pH 8.3, 192 mM Glycine and 20% (v/v) ethanol]. Blots were blocked for 30 min with 5% BSA in Tris-buffered saline (TBS) and incubated overnight at 4°C with the primary antibody diluted in 5% BSA in TBS with Tween-20 (TBS-t). Secondary antibodies, conjugated to horseradish peroxidase (HRP), were incubated with the blot for 45 min at room temperature. Between antibody incubations, blots were washed three times with TBS-t. As final step before imaging, blots were washed in TBS. HRP signals were visualized using enhanced chemiluminescence (ECL) detection (SuperSignal West Pico PLUS, Thermo Fisher Scientific, cat. no. 34580) and imaged with a ImageQuant LAS 4000 (GE Healthcare) machine. Intensities of bands were quantified using the Gel Analyzer plugin in ImageJ. Band intensity is corrected for background and normalized to the loading control. See Fig. S5 for an overview of uncropped western blots from representative examples.

### Statistical analysis

Data was analyzed using Microsoft Excel and statistical analysis was performed using Prism Graphpad V6. All bar graphs represent the mean±s.e.m. Violin plots highlight median and quartiles. Data was checked for normal distribution using the D'Agostino-Pearson normality test. In case of normal distribution, a two-tailed paired Student's *t*-test was used when two groups were compared. One-way or two-way analysis of variance (ANOVA) with post tests as denoted in figure legends was used to compare two or more groups. Respective statistical tests are mentioned in figure description. *P*-values are indicated by asterisks and defined as: ns, not significant; *P<0.05; **P<0.01; ***P<0.001; ****P<0.0001.

## Supplementary Material



10.1242/joces.261687_sup1 Supplementary information

## Data Availability

All relevant data can be found within the article and its supplementary information.
