# Supplementary material for: DLC1 promotes mechanotransductive feedback for YAP via RhoGAP-mediated focal adhesion turnover
Source: J Cell Sci. 2024 Apr 30;137(8):jcs261687. doi: 10.1242/jcs.261687 (PMC11112125; doi:10.1242/jcs.261687)
Supplement: Supplementary information [file joces-137-261687-s1.pdf]

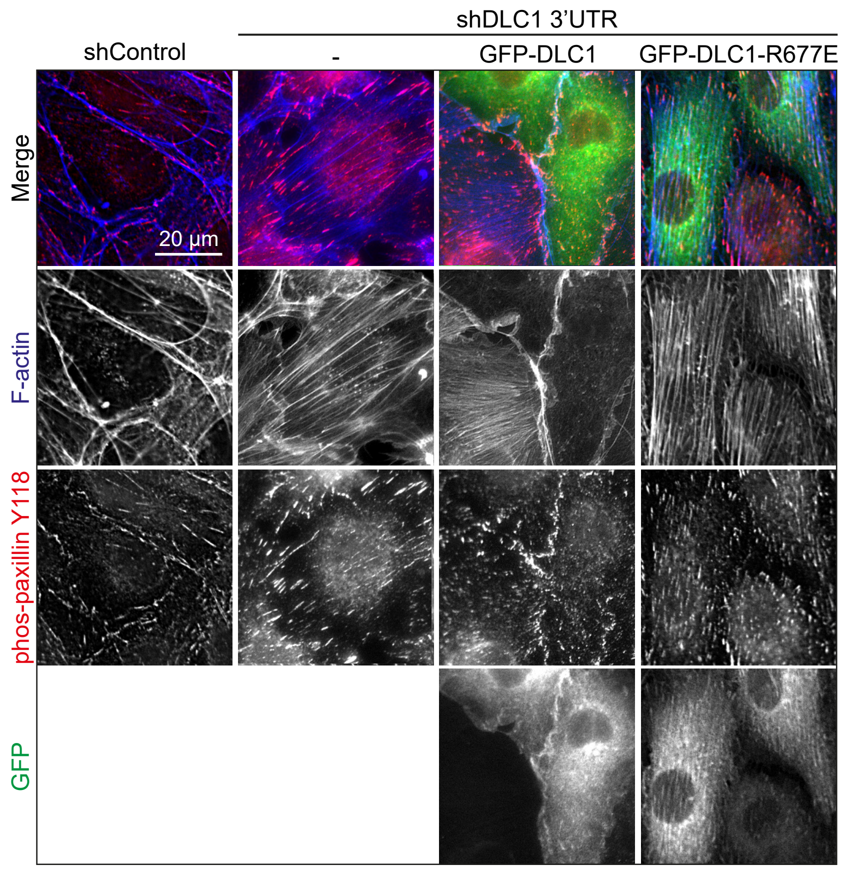

Fig. S1. Representative immunofluorescence images of HUVECs transduced with shControl, shDLC1 3'UTR and subsequently rescued with GFP-DLC1 or GFP-DLC1-R677E (green) and stained for F-actin (blue) and phospho-paxillin Y118 (red).

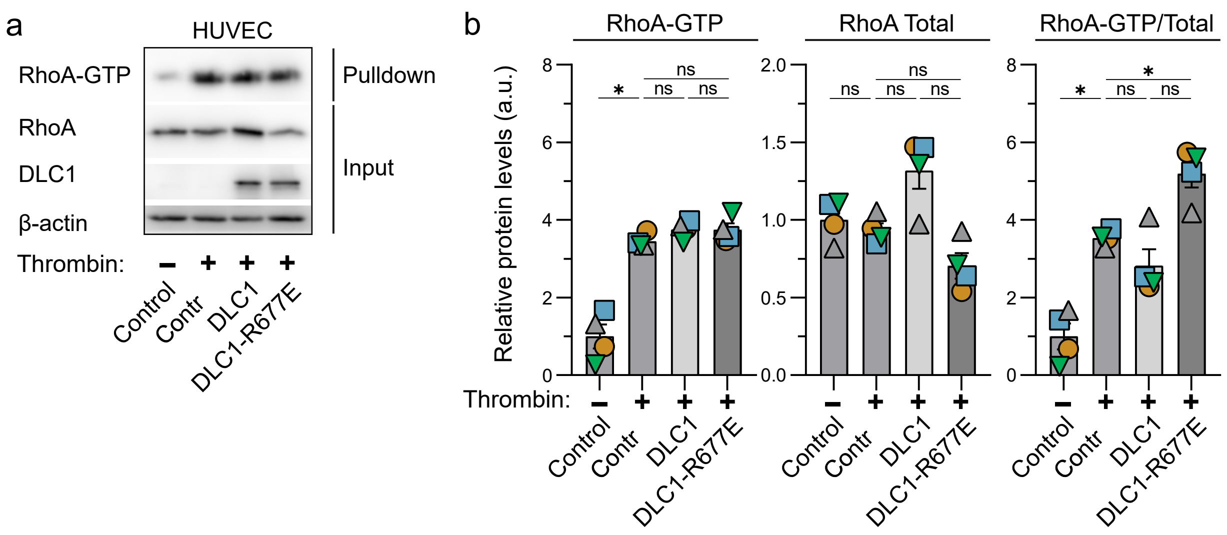

Fig. S2. (a) Representative Western blot analysis of RhoA-GTP levels in lysates from HUVECs transduced with GFP (Control), GFP-DLC1 or GFP-DLC1-R677E and stimulated with thrombin for 2 minutes from rhotekin pull-downs. Blotted for RhoA, DLC1 and β-actin (loading control). **(b)** Bar graphs show RhoA-GTP levels, RhoA levels and the ratio between RhoA-GTP and RhoA levels upon thrombin treatment. Data is from n=4 independent experiments; one-way ANOVA, Tukey's multiple comparisons test.

Journal of Cell Science • Supplementary information

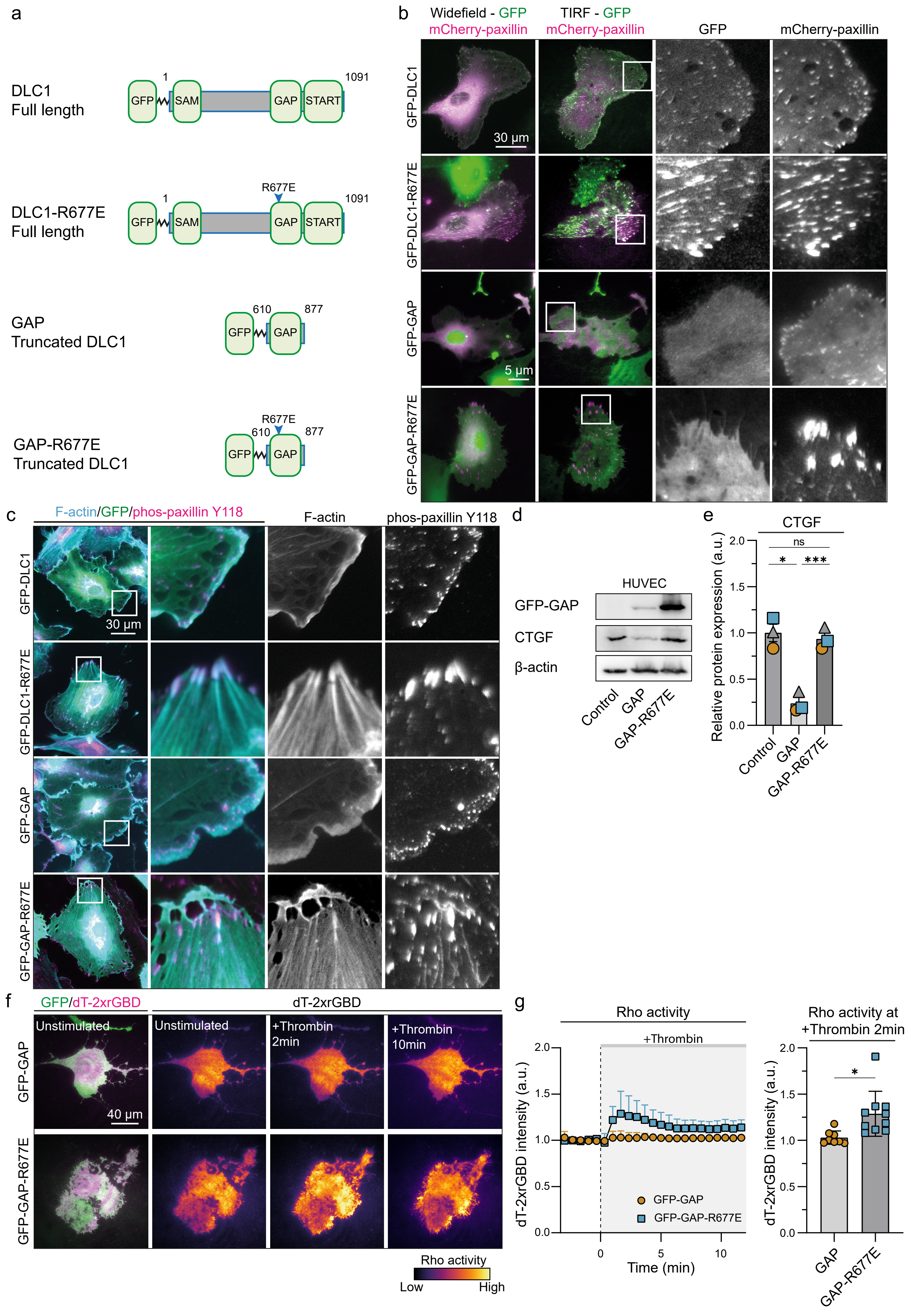

Journal of Cell Science • Supplementary information

Fig. S3.

(a) Schematic overview of the full length DLC1 and the truncated GAP domain mutant and their catalytic inactive R677E variants. **(b)** Representative Widefield and TIRF images from live imaged HUVECs transduced with GFP-DLC1, GFP-DLC1-R677E, GFP-GAP or GFP-GAP-R677E and mCherry-paxillin. **(c)** Representative immunofluorescence images of HUVECs expressing GFP-DLC1, GFP-DLC1-R677E, GFP-GAP or GFP-GAP-R677E (green) and stained for F-actin (cyan) and phosphorylated paxillin Y118 (magenta). **(d)** Representative Western blot analysis of lysates from HUVECs transduced with GFP (Control), GFP-GAP or GFP-GAP-R677E. Blotted for GFP, CTGF and β-actin (loading control). **(e)** Bar graph shows quantified CTGF levels. Data is from n=3 independent experiments; one-way ANOVA, Tukey's multiple comparisons test. **(f)** Representative TIRF images from live imaged HUVECs stimulated with thrombin and co-expressing Rho biosensor dT-2xrGBD with GFP-GAP or GFP-GAP-R677E. See corresponding Movie 3 for the 15 minutes time-lapse recording. **(g)** Normalized fluorescence intensity of the Rho biosensor dT-2xrGBD upon thrombin stimulation over time and at 2 minutes of stimulation. Data is from n=3 independent experiments, GAP = 8 cells, GAP-R677E = 10 cells; two-tailed t-test.

Journal of Cell Science • Supplementary information

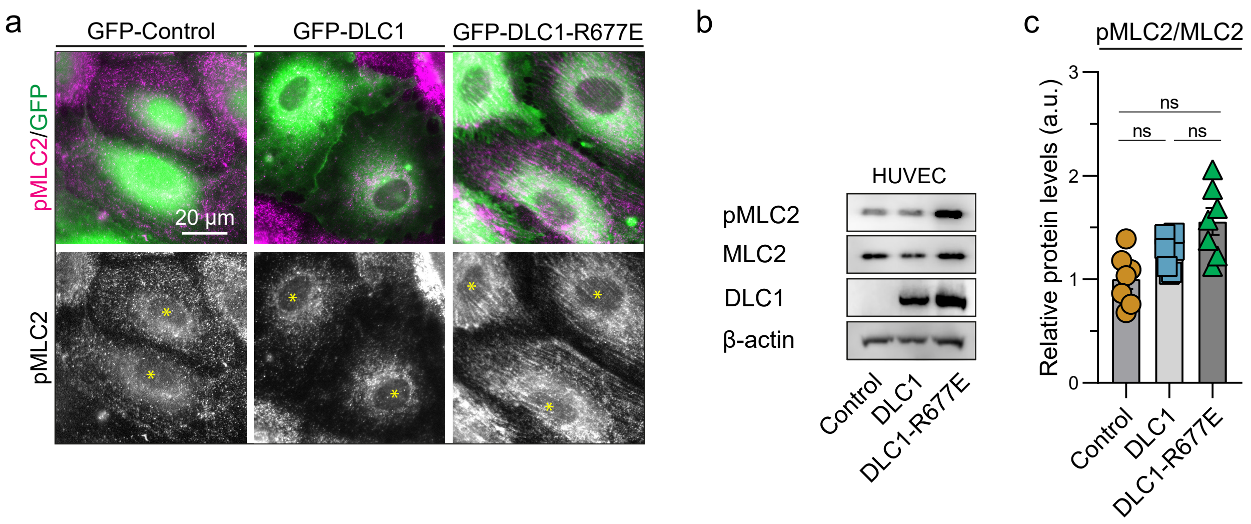

Fig. S4.

(a) Representative immunofluorescence images of HUVECs transduced with GFP (Control), GFP-DLC1 or GFP-DLC1-R677E (green) and stained for phospho-MLC2 S19 (pMLC2; magenta). **(b)** Western blot analysis of lysates from HUVECs transduced with GFP (Control), GFP-DLC1 or GFP-DLC1-R677E. Blotted for phospho-MLC2 S19 (pMLC2), MLC2, DLC1 and β-actin (loading control).

(c) Bar graph shows the ratio between pMLC2 and MLC2. Data is from n=7 independent experiments; one-way ANOVA, Tukey's multiple comparisons test.

Journal of Cell Science • Supplementary information

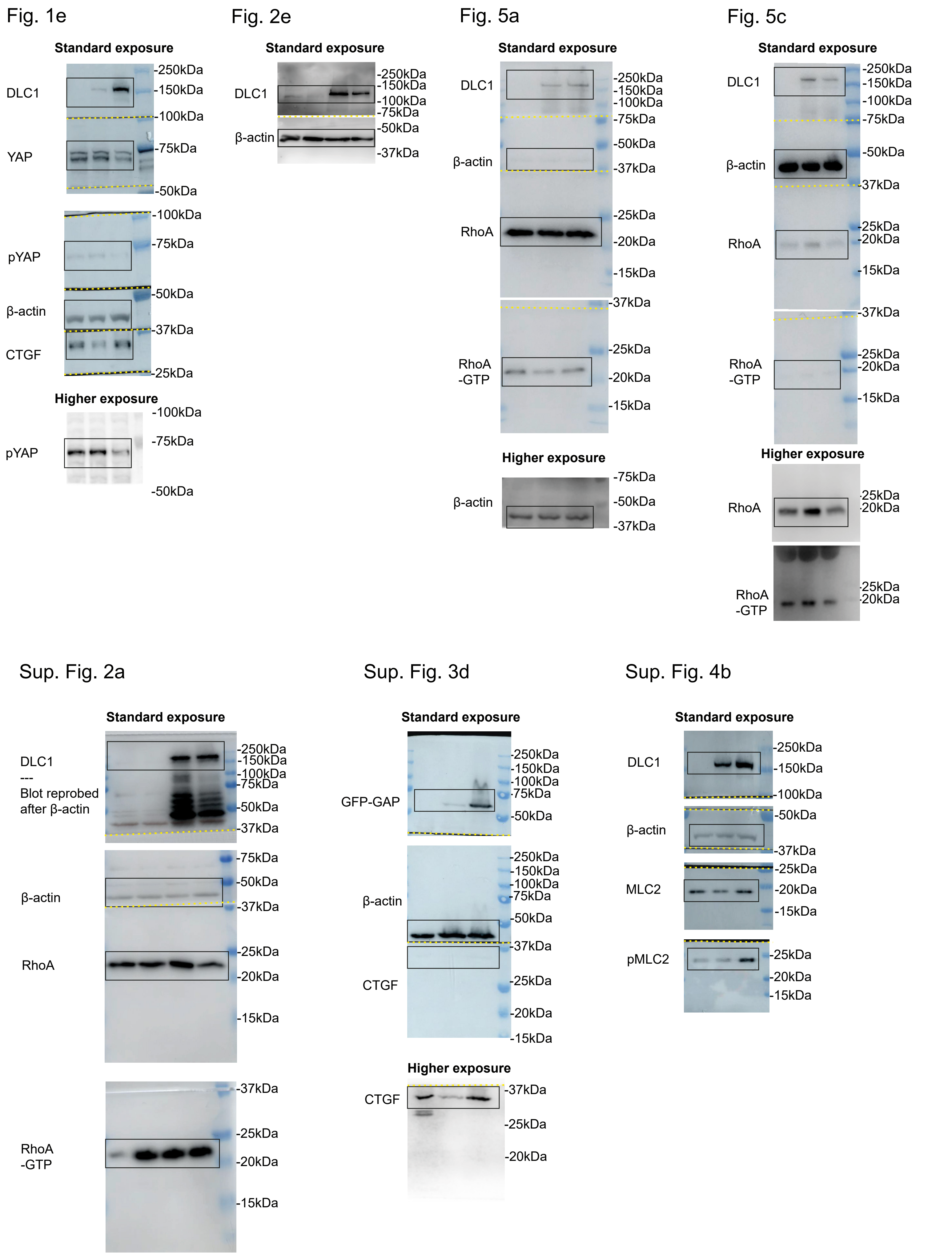

Fig. S5. Overview of total Western Blots from representative examples.

Journal of Cell Science • Supplementary information

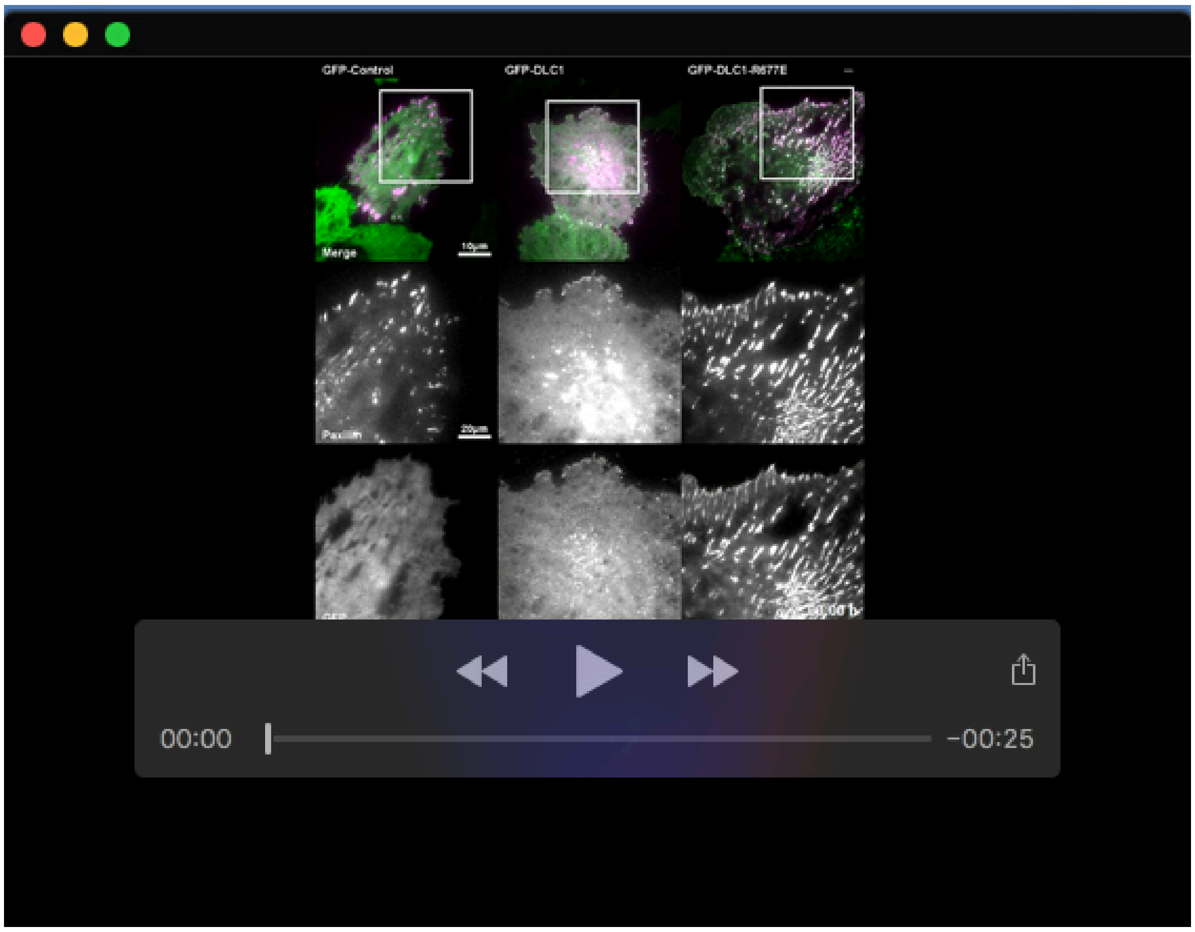

Movie 1. DLC1 controls focal adhesion turnover and maturation in a RhoGAP-dependent manner.
Live TIRF microscopy recordings of imaged HUVECs transduced with GFP (Control), GFP-DLC1 or GFP-DLC1-R677E and mCherry-paxillin.

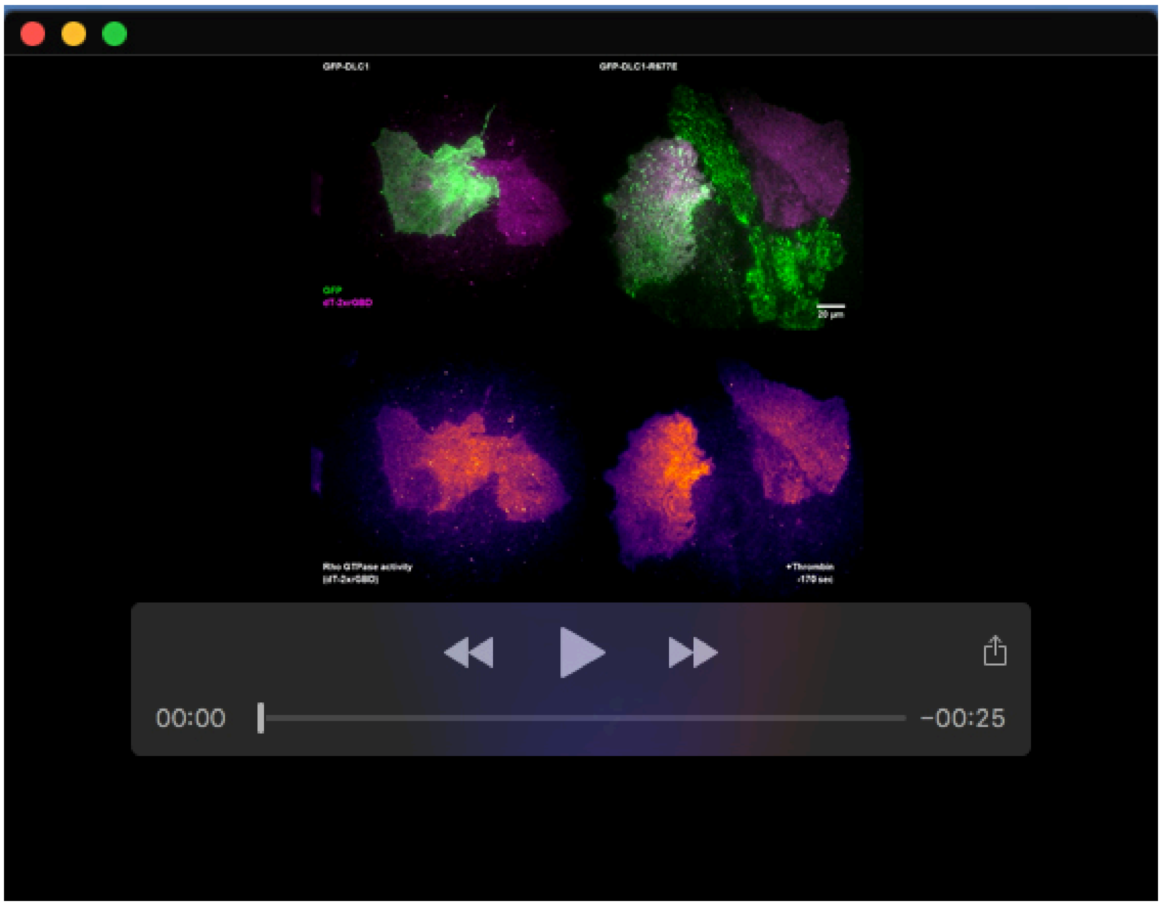

Movie 2. The RhoGAP function of DLC1 inhibits basal Rho signaling.
Live TIRF microscopy recordings of imaged HUVECs stimulated with thrombin and expressing the Rho biosensor dT-2xrGBD in control cells, or in GFP-DLC1 or GFP-DLC1-R677E cells.

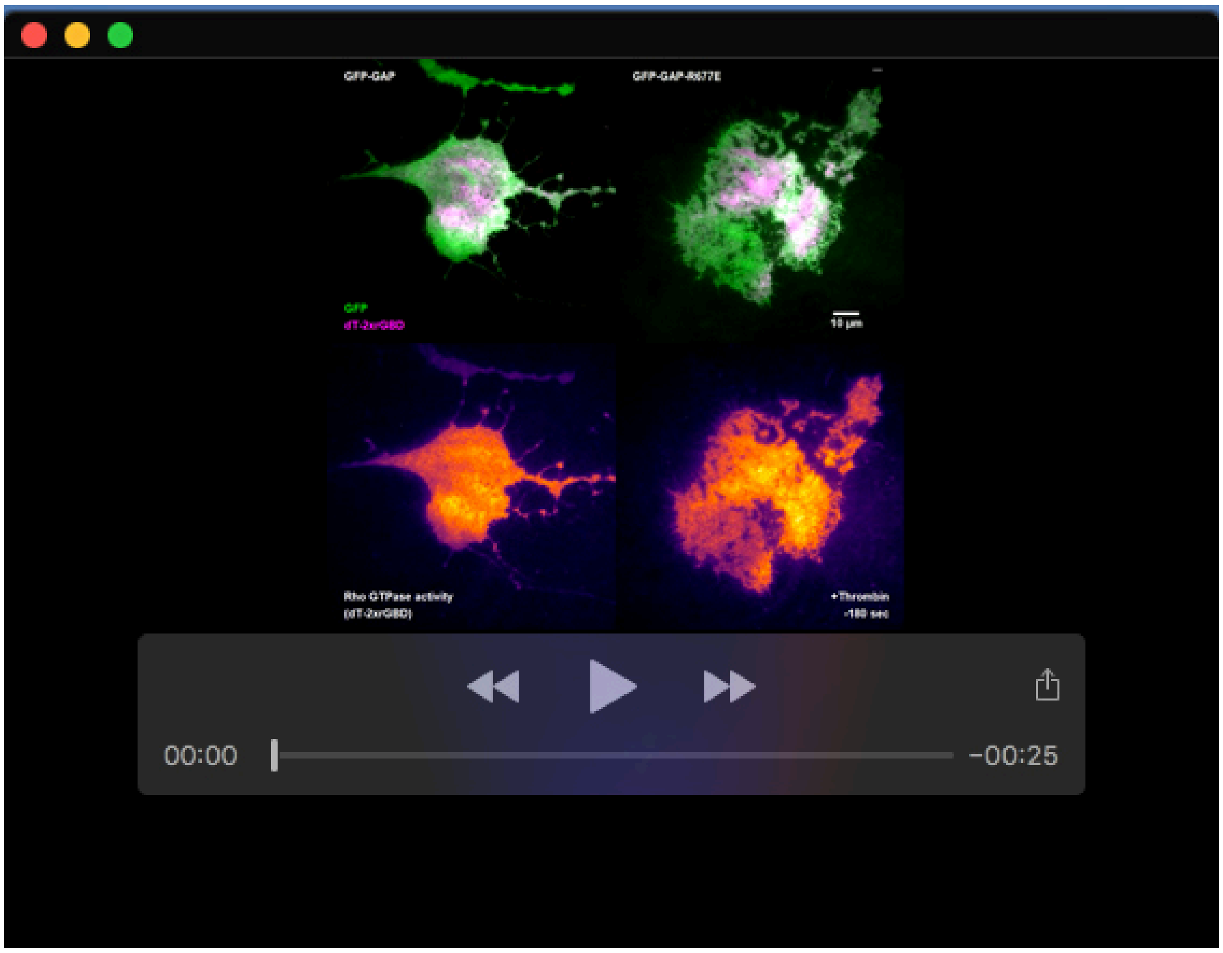

Movie 3. The RhoGAP domain of DLC1 inhibits basal Rho signaling.
Live TIRF microscopy recordings of imaged HUVECs stimulated with thrombin and expressing the Rho biosensor dT-2xrGBD in control cells, or in GFP-GAP or GFP-GAP-R677E cells.

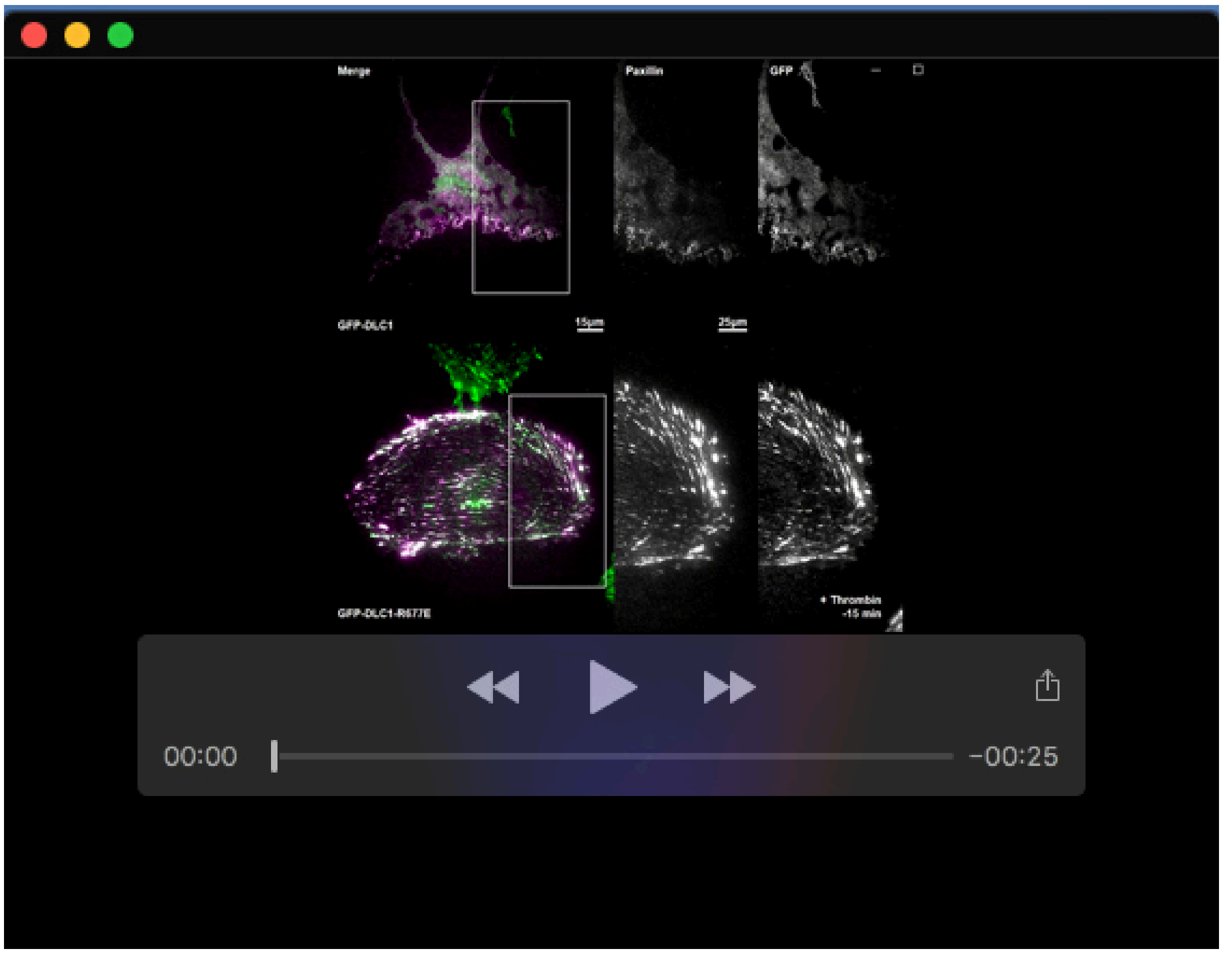

Movie 4. Thrombin restores cell adhesion in DLC1-overexpressing HUVECs.
Live TIRF microscopy recordings of imaged HUVECs transduced with GFP (Control), GFP-DLC1 or GFP-DLC1-R677E and mCherry-paxillin stimulated with thrombin.
