# Supplementary material for: DLC1 promotes mechanotransductive feedback for YAP via RhoGAP-mediated focal adhesion turnover
Source: J Cell Sci. 2024 Apr 30;137(8):jcs261687. doi: 10.1242/jcs.261687 (PMC11112125; doi:10.1242/jcs.261687)
Supplement: Supplementary file 1 [file joces-137-261687_review_history.pdf]

DLC1 promotes mechanotransductive feedback for YAP via RhoGAP-mediated focal adhesion turnover

Aukie Hooglugt, Miesje M. van der Stoel, Apeksha Shapeti, Beau F. Neep, Annett de Haan, Hans van Oosterwyck, Reinier A. Boon and Stephan Huveneers
DOI: 10.1242/jcs.261687

Editor: Kairbaan Hodivala-Dilke

Review timeline
Original submission: 28 September 2023
Editorial decision: 7 November 2023
First revision received: 19 February 2024
Accepted: 25 March 2024

Original submission

First decision letter

MS ID#: JOCES/2023/261687

MS TITLE: DLC1 promotes mechanotransductive feedback for YAP via RhoGAP-mediated focal adhesion turnover

AUTHORS: Aukie Hooglugt, Miesje M. van der Stoel, Apeksha Shapeti, Beau Neep, Annett de Haan, Hans van Oosterwyck, Reinier A Boon, and Stephan Huveneers

ARTICLE TYPE: Research Article

We have now reached a decision on the above manuscript.

To see the reviewers' reports and a copy of this decision letter, please go to: https://submit-jcs.biologists.org and click on the 'Manuscripts with Decisions' queue in the Author Area. (Corresponding author only has access to reviews.)

As you will see, the reviewers raise a number of substantial criticisms that prevent me from accepting the paper at this stage. They suggest, however, that a revised version might prove acceptable, if you can address their concerns. If you think that you can deal satisfactorily with the criticisms on revision, I would be pleased to see a revised manuscript. We would then return it to the reviewers.

Please ensure that you clearly highlight all changes made in the revised manuscript. Please avoid using 'Tracked changes' in Word files as these are lost in PDF conversion.

I should be grateful if you would also provide a point-by-point response detailing how you have dealt with the points raised by the reviewers in the 'Response to Reviewers' box. Please attend to all of the reviewers' comments. If you do not agree with any of their criticisms or suggestions please explain clearly why this is so.

Reviewer 1

Advance summary and potential significance to field

© 2024. Published by The Company of Biologists under the terms of the Creative Commons Attribution License (https://creativecommons.org/licenses/by/4.0/).

Previous work from the authors demonstrated that DLC1 is a downstream target of YAP/TAZ transcriptional control of focal adhesion disassembly, cell polarisation, migration and angiogenic sprouting.
In the present study, the authors demonstrate that these effects are dependent of DLC1-GAP activity.
Although the role of DLC1 on destabilising focal adhesions and F-actin fibers has been described previously the authors identify a dominant negative effect of DLC1 on YAP translocation to the nucleus, implying possibly a DLC1 -GAP domain effect.

Comments for the author

The presented data supports that DLC1 through the GAP domain destabilises F-actin fibers and focal adhesions, whereas the effect on Rho activity and actomyosin contractility is weak.
It is not clear why the authors analyse YAP translocation to the nucleus upon thrombin-induced stabilisation of FAs to support the role of DLC1 as a negative regulator of YAP only in DLC1 expressing HUVECs and do not include the DLC1-GAP mutant. These experiments should also be performed using the DLC1-GAP mutant to better support the title of the paper: “DLC1 promotes mechanotransductive feedback for YAP via RhoGAP-mediated focal adhesion turnover”.
Also, the authors should provide more accurate description of the data related to figures 4 and 5 and revised appropriately their model (Rho arrows and intracellular tension due to actomyosin contractility).
Additionally, the authors should address confusing repetitions or misleading statements in the text of the manuscript.
1. In the concluding paragraph in the introduction, the authors should state clearly what is the advancement of the paper over the previous work of the lab and others. The concluding sentence: “Importantly, we provide evidence that DLC1 expression restricts focal adhesion maturation by decreasing Rho/ROCK-mediated intracellular tension at the integrin-ECM adhesion interface” refers to previously published data by the authors (van der Stoel, JCS 2020).

2. in the first paragraph of the results the authors state “To investigate whether DLC1 controls YAP functions, we overexpressed GFP-tagged DLC1 (GFP-DLC1) or free GFP (Control) in Human Umbilical Vein Endothelial Cells (HUVECs) and examined YAP localization using immunofluorescence analysis.” But they describe control experiments and then they repeat: “Next, we investigated the effect of DLC1 overexpression on YAP localization.” before describing the overexpression results.

3. In the description of fig 2a, the authors state that overexpression of DLC1 in HUVECs resulted in focal adhesions that failed to mature and the contrary is true for DLC1-GAP mutant. However, the FA marker that they use is p-paxillin, which does not label mature adhesions. Similarly, the quantification provided is FAs/um3 and not mature FAs vs immature or central FAs vs peripheral. The above statement is better supported by the following experiments, e.g. live imaging and measurement of lifetime of FAs.

4. The statement in the results describing the HUVEC sprouting :“This suggests that their might be a difference in basal cell-cell versus cell-ECM binding efficiency” is not supported by any experimental data and should be removed.

5. The statement in the results examining the effect of DLC1 overexpression in the Rho-GTP activity :
“These findings indicate that DLC1 overexpression in endothelial cells modulates RhoA, and potentially RhoB GTPase signaling in a GAP-dependent manner” is not supported by the data, because the authors data show that the ratio of Rho-GTP/ total Rho levels is the same in control, DLC1 overexpression and DLC1-GAP mutant HUVECs. The effect of increased Rho-activity in DLC1 overexpressed cells and the lack of increased Rho-activity in DLC1-GAP mutant HUVECs is the result of increased total Rho levels. The lack of an effect on Rho-GTP activity agrees with the thrombin data presented in fig 5 and previous data from the same lab showing no effect in Rho-activity upon DLC1 knockdown. Therefore, the graph 3e is misleading and should be removed together with the related statements in the results and discussion.

© 2024. Published by The Company of Biologists under the terms of the Creative Commons Attribution License (https://creativecommons.org/licenses/by/4.0/).

6. To determine whether there is an increase in P-MLC in DLC1-GAP mutant cells, given the increased total MLC levels, the ratio of P-MLC/MLC should be calculated. The increased of pMLC immunofluorescent could be a secondary effect of stabilised FAs and not a primary effect of DLC1-GAP activity.
7. In legend of figure 4 there is the statement: “Cells without any measureable tractions were not included in this quantification.” However, in graph 4j, there are many zero values on DLC1 and DLC1-GAP mutant cells.
The data in the graph should be corrected. Furthermore, the colour scale of traction forces should be corrected to depict better the data. As it is now, it shows that cells with zero traction forces are spread.
8. The authors should discuss the differences between the overexpression of the dominant negative DLC1-GAP mutant and the DLC1 knockdown published before (van der Stoel) on focal adhesion assembly/disassembly rates and the lifetime of focal adhesions.
9. The authors should discuss how the reduced F-actin formation and FA stabilisation leading to reduced YAP translocation increase in vitro endothelial sprouting.

Additional minor points:
1. In the quantification of TIRF experiments, indicate how many FAs were analysed per cell in the different conditions.
2. In the mosaic experiments, the authors should quantify the number of spheroids having control cells vs DLC1 and DLC1-GAP mutant cells in the periphery at the onset of VEGF stimulation. Also, the length of each sprout from RFP control and GFP-DLC1, DLC1-GAP mosaic spheroids should be calculated instead of the cumulative sprout length. This quantification would indicate better the defect in VEGF-stimulated endothelial cell migration.

<u>Reviewer 2</u>

Advance summary and potential significance to field

This exciting study evaluates the role of the RhoA GAP, DLC1, in endothelial cell migration and angiogenesis, via feedback regulation of the transcriptional regulator YAP. This is an elegant and focused study that provides critical mechanistic insight into both the mediators of the feedback loop previously discovered by the team and provides new insights into DLC1 biochemistry, and the mechanobiology of focal adhesion regulation. The authors characterize the RhoGAP activity of DLC1 and its effects on YAP nuclear translocation and transcriptional activity, focal adhesion dynamics, Rho-ROCK signaling, and cytoskeletal tension.

This work advances our understanding of endothelial migration regulation and establishes DLC1 activity as a crucial negative feedback mediator, downstream of YAP, which acts to modulate YAP/TAZ activity to regulate sprouting angiogenesis.
The results support their conclusions by demonstrating that signaling through DLC1's RhoGAP domain leads to a reduction in the nuclear localization of YAP and its transcriptional activity, which influences YAP-mediated cellular processes such as regulating focal adhesion turnover, modulating traction forces, and facilitating sprouting angiogenesis. An exciting advance, this paper will be of great interest to the broad readership of the JCS and will be valuable to readers with interests in cytoskeletal and focal adhesion regulation, mechanobiology, and vascular biology.

Enthusiasm for the contributions of the paper is moderated by a rather confusing presentation. Increasing the clarity of the paper for this broad audience, who may not be familiar with the group's prior papers on YAP/TAZ-DLC1 feedback, will increase both its reach and impact. We provide some suggestions toward this end below, but emphasize the importance and quality of the paper and its relevance for the JCS community.

Comments for the author

• This work builds the authors' previous discovery that Deleted-in-Liver-Cancer-1 (DLC1) is a direct target of the YAP mechanotransducer and promotes sprouting angiogenesis through a mechanotransductive feedback loop that regulates cytoskeletal contractility. The prior literature

© 2024. Published by The Company of Biologists under the terms of the Creative Commons Attribution License (https://creativecommons.org/licenses/by/4.0/).

on this YAP-RhoA feedback loop while mentioned in the introduction, are not sufficiently detailed for a general audience. Understanding these previous publications is key to understanding the starting point and context of the current work. The authors should make it easier for the reader to make this connection by adding a summary of the previous results in the introduction - because this is a feedback loop, the reader can become easily confused without clear description of where to start. We also suggest including a schematic as Figure 1 to illustrate what is currently known about the feedback loop and to identify the knowledge gap that will be addressed in this work.

- The authors aimed “to investigate the role of DLC1 in the modulation of YAP activity in endothelial cells during angiogenic sprouting”. The way the manuscript is currently structured makes it difficult to follow. We recommend a re-organization of the paper into two parts. Part 1 would focus on the role of DLC1 in the modulation of YAP activity [Figures 3, 1], establishing the GAP activity of DLC1 as a mediator of the YAP-RhoA feedback loop. Part 2 would then address the key gap of the paper, namely how does the RhoGAP activity of DLC1 function in regulation of cell contractility, tension generation, focal adhesion turnover, and functional sprouting [Figures 2, 4, 5].
- The results from RhoA and RhoA-GTP quantification in HUVECs were confusing. For the aim of this paper, it would be unnecessary to do more experiments to understand these observations; however, the authors should make it clear why these results are unexpected and what they might mean. This is especially important because it is the basis for understanding the thrombin stimulation experiment (Figure 5b, c). Because this confusing result is the basis for the following argument: “DLC1 does not regulate global Rho signaling, but may control local intracellular contractility at focal adhesions,” it limits confidence in this conclusion. While it is clear from the data that DLC1 GAP activity regulates focal adhesions, the data do not directly show local DLC1 or RhoA activity IN or ON the focal adhesions. Because it is hard to interpret the global RhoA-GTP blot data, it is hard for us to follow the argument from the thrombin treatment experiment. This is further complicated because Thrombin is not a direct RhoA-agonist, but has a variety of signaling consequences in endothelial cells downstream of PAR-1).
- The concluding remarks focus on the role of DLC1 in cancer. The authors have not previously mentioned cancer in the manuscript and it takes focus away from the aim of the paper. We suggest rewriting or removing this section.
- Discuss the potential roles of TAZ vs. YAP.
- The authors “observed many long-lived stationary focal adhesions in the cell body that were not detected by FAAS”. Expand on the limitations of measuring focal adhesions using FAAS and how that impacts your results. Is there a way to measure these long-lasting FA's?
- Thrombin is not a specific RhoA activator. How does this impact your results and interpretation? Explanation of why thrombin was selected and what results were expected could help your readers follow the reasoning.

Reviewer 3

Advance summary and potential significance to field

The report by Hooglugt and colleagues describes the role of DLC1 in the regulation of YAP nuclear localization, in a process mediated by a RhoA/ROCK signaling pathway. The authors describe a negative feedback loop, by which DLC1 inhibits YAP’s nuclear localization, in particular during cell migration and angiogenesis. The process requires the GAP activity and it is proposed to work through RhoA/ROCK.
The study is significant as it describes a novel role for DLC1/YAP and may explain tight regulation required that is required to control angiogenesis.

Comments for the author

The experiments are well designed for the most part and the results are solid in general. However, there are two important aspects of this study that, in my opinion, need to be revised to further support the conclusions made by the authors:
First, most experiments are based on results from overexpression of a RhoGAP. The question is whether by grossly overexpression a RhoGAP, the effects observed are due to a global, non-specific inactivation of RhoA in the cell. Second, there is uncertainty, and also lack of clarity, regarding

© 2024. Published by The Company of Biologists under the terms of the Creative Commons Attribution License (https://creativecommons.org/licenses/by/4.0/).

whether DLC1 modulates RhoA activity locally, or another RhoGTPase in HUVECS, as no inhibitions of RhoA is observed in DLC1 overexpressing cells.
Addressing these concerns will provide a clearer picture of the pathway involved and will strengthen the manuscript significantly.

Specific comments:
-The role of RhoA downstream DLC1 is the weakest aspect of this study. The experiments showing that DLC1 overexpression does not inactivate RhoA in HUVECs are confusing. It is hard to understand how high total RhoA-GTP in DLC1 overexpressing cells correlates with less actin polymerization. The authors also state that RhoB may be affected but the differences in expression levels are not significantly different and the activity was not tested. It may be possible that the activity of RhoB and RhoC are decreased and that could explain the effects on YAP activation. My recommendation is to perform additional experiments to confirm or rule out the direct role of RhoA or other related GTPases in the regulations of the events downstream of DLC1. 1) Measure the local inactivation of RhoA using a RhoA sensor. I still believe that the most likely possibility is global inactivation of RhoA/B or C based on the high levels of overexpression, but the authors propose local inactivation of RhoA by DLC1. A Rho sensor should be able to distinguish between these scenarios. 2) Measure the activation levels of RhoB and C. RhoC appears to be relevant as it has been shown to regulate YAP through the function of another GAP ARHGAP18 (https://doi.org/10.1186/s12964-020-0511-7).
-A second key concern is the level of overexpression of DLC1 and DLC1 mutants. In particular since these are not stably expressing cell lines, the levels of overexpression (or rescue) detected by WB correspond only to a fraction of the cells, so they may be even higher on a per cell basis. For example, in Supp. Fig. 1h there are probably several fold overexpression levels in the KD/rescue experiment. At these levels of overexpression, the question is whether the effects observed are specific to DLC1 function, or to a non-specific global inhibition of RhoA activity in cells. This can be tested by overexpressing a non-related RhoA GAP (or just the GAP domain of DLC1). If the effects are non-specific for DLC1 then the non-specific GAP should not phenocopy DLC1 overexpression.
-One concern in general regarding the overexpression experiments is that he levels of transfection efficiency (at least in the pictures shown) seems to vary a lot. This is not a problem for immunofluorescence, but it may represent a problem in the biochemical assays, because depending on the efficiency of transfection, the effects may be more or less pronounced (see for example the difference of expression levels between DLC1 and 677 in Fig. 1i. This could represent differences of transfection efficiency, expression/stability differences between WT and 677, or a combination of both.
-There are complementary experiments silencing the expression of DLC1, which I believe are more physiologically relevant than the overexpression ones. However, in this manuscript, they are all presented as Supplemental Figures and the emhasis is placed on the overexpression experiments. I believe that some, or all of them should be in the main figures.
-It is hard to judge a result like the one in Supp. Fig. 2a without quantification and replication. Some panels show just one cell. Maybe FA number and/or size could be quantified as done in Fig 2. Similarly, quantification of spreading and focal adhesion dynamic changes (or lack thereof) should be provided to support the conclusion in Fig. 5a and Supp. 5a.

First revision

Author response to reviewers' comments

We thank all three reviewers for their positive evaluation, their time to carefully review our manuscript and for the valuable comments. We have incorporated their suggestions and performed new experiments to address remaining questions regarding the effect of DLC1 on Rho activity using a translocation-based fluorescence biosensor and truncated DLC1 variants. Please find below our point- by-point answers to the reviewers.

Reviewer 1 Advance Summary and Potential Significance to Field:
Previous work from the authors demonstrated that DLC1 is a downstream target of YAP/TAZ

© 2024. Published by The Company of Biologists under the terms of the Creative Commons Attribution License (https://creativecommons.org/licenses/by/4.0/).

transcriptional control of focal adhesion disassembly, cell polarisation, migration and angiogenic sprouting. In the present study, the authors demonstrate that these effects are dependent of DLC1-GAP activity. Although the role of DLC1 on destabilising focal adhesions and F-actin fibers has been described previously, the authors identify a dominant negative effect of DLC1 on YAP translocation to the nucleus, implying possibly a DLC1 -GAP domain effect.

Reviewer 1 Comments for the Author:

1. General remarks

The presented data supports that DLC1 through the GAP domain destabilises F-actin fibers and focal adhesions, whereas the effect on Rho activity and actomyosin contractility is weak. It is not clear why the authors analyse YAP translocation to the nucleus upon thrombin-induced stabilisation of FAs to support the role of DLC1 as a negative regulator of YAP only in DLC1 expressing HUVECs and do not include the DLC1-GAP mutant. These experiments should also be performed using the DLC1-GAP mutant to better support the title of the paper: “DLC1 promotes mechanotransductive feedback for YAP via RhoGAP- mediated focal adhesion turnover”.

We apologize for not explaining the rationale for this experiment sufficiently clear. In the experiments presented in Figures 1, 2 and 4 we have indeed compared-side-by side the effect of DLC1 versus DLC1- R677E (GAP dead) on YAP translocation and focal adhesion turnover. These experiments already establish that DLC1 expression promotes mechanotransductive feedback for YAP via RhoGAP-mediated focal adhesion turnover. The purpose of the concluding experiments in Figure 6 are to investigate whether the DLC1-driven negative feedback pathway could still bypassed by inducing overall Rho- mediated contractility, thereby establishing whether this feedback loop is still active and subject to regulation. Thrombin is a well-known activator of RhoA and actomyosin-mediated contractility in endothelial cells (Amerongen et al, 2000, Botros et al, 2020, Huveneers et al, JCB, 2012). Our experiments demonstrate that thrombin-treatment activates overall RhoA-GTP loading (Supplemental Figure 2) and restores nuclear YAP levels in DLC1 overexpressing sparse cells (Figure 6c, d). There is no rationale to add thrombin to DLC1-R677E expressing cells as the DLC1-mediated negative feedback towards YAP is not active in these cells (Figure 1, 2). Instead, as control for the experiment, we included the GFP expressing HUVECs to validate that thrombin induced endothelial contractility and YAP nuclear translocation within the timeframe of the experiment. We have now better emphasized these aspects in the results section of the revised manuscript.

Of note, we concur with the reviewer that the evidence for a direct role of DLC1 on Rho signaling was not yet fully supported in the first manuscript. We have now included new experiments to address this, by using a translocation-based fluorescence biosensor and TIRF microscopy of the basal adhesion plane of the endothelial cells. We expressed this sensor in combination with both DLC1-GFP and DLC1-R677E- GFP in primary endothelial cells. Following subsequent treatment with thrombin, we find clear induction of Rho activation in control cells and DLC1-R677E expressing cells. Confirming that (i), thrombin induces Rho activation in control conditions, and (ii) that Rho activation occurs to similar extent in cells that express the GAP dead DLC1 variant. Intriguingly, in DLC1 expressing cells, we find that thrombin could not activate Rho at the basal membrane (new Figure 5e, f), whereas overall Rho GTP-loading still occurs (Supplemental Figure 2) in these cells and is sufficient to rescue YAP translocation (Figure 6c, d).

Also, the authors should provide more accurate description of the data related to figures 4 and 5 and revised appropriately their model (Rho arrows and intracellular tension due to actomyosin contractility). Additionally, the authors should address confusing repetitions or misleading statements in the text of the manuscript.

Agreed, we have adjusted the manuscript accordingly by removing repetitions and thereby improved the readability of the text. We have also adjusted the content in these figures, the model and the corresponding text to present the data as accurate and clear as possible for readers. See applied changes in the results section of the revision.

2. Major points

1. In the concluding paragraph in the introduction, the authors should state clearly what is the

© 2024. Published by The Company of Biologists under the terms of the Creative Commons Attribution License (https://creativecommons.org/licenses/by/4.0/).

advancement of the paper over the previous work of the lab and others. The concluding sentence: "Importantly, we provide evidence that DLC1 expression restricts focal adhesion maturation by decreasing Rho/ROCK-mediated intracellular tension at the integrin-ECM adhesion interface" refers to previously published data by the authors (van der Stoel, JCS 2020).
We apparently did not do a great job in stating the advancement of our current findings above what is already known. We have adjusted the text to more clearly state the advancement of the paper over previous studies and our specific previous findings, please see marked changes in the introductory text. The concluding paragraph now reads as follows:

"In the present study, we uncover a negative feedback loop between DLC1 expression and YAP activity during collective migration and sprouting angiogenesis. Our results reveal that DLC1, functioning as a YAP effector, in turn reduces YAP transcriptional activity and promotes angiogenic sprouting in a RhoGAP-dependent manner. DLC1 GAP activity prevents basal Rho signaling, thereby limiting cytoskeletal forces at the integrin-ECM adhesion interface. Collectively, these findings underscore the significance of DLC1 in modulating Rho signaling for mechanotransductive feedback to properly control YAP activity during sprouting angiogenesis.."

2. in the first paragraph of the results the authors state "To investigate whether DLC1 controls YAP functions, we overexpressed GFP-tagged DLC1 (GFP-DLC1) or free GFP (Control) in Human Umbilical Vein Endothelial Cells (HUVECs) and examined YAP localization using immunofluorescence analysis." But they describe control experiments and then they repeat: "Next, we investigated the effect of DLC1 overexpression on YAP localization." before describing the overexpression results.
The revised manuscript has been adjusted throughout accordingly.

3. In the description of fig 2a, the authors state that overexpression of DLC1 in HUVECs resulted in focal adhesions that failed to mature and the contrary is true for DLC1-GAP mutant. However, the FA marker that they use is p-paxillin, which does not label mature adhesions. Similarly, the quantification provided is FAs/um3 and not mature FAs vs immature or central FAs vs peripheral. The above statement is better supported by the following experiments, e.g. live imaging and measurement of lifetime of FAs.
Indeed we used the phosphorylated paxillin stainings to label all integrin-based adhesions, not specifically mature focal adhesions. We now restricted the results description of the immunostaining to the number of focal adhesions per um^2, based on the corresponding quantifications (Figure 4a, b).

4. The statement in the results describing the HUVEC sprouting :"This suggests that their might be a difference in basal cell-cell versus cell-ECM binding efficiency" is not supported by any experimental data and should be removed.
Agreed, the sentence is deleted.

5. The statement in the results examining the effect of DLC1 overexpression in the Rho-GTP activity: "These findings indicate that DLC1 overexpression in endothelial cells modulates RhoA, and potentially RhoB, GTPase signaling in a GAP-dependent manner" is not supported by the data, because the authors data show that the ratio of Rho-GTP/ total Rho levels is the same in control, DLC1 overexpression and DLC1-GAP mutant HUVECs. The effect of increased Rho-activity in DLC1 overexpressed cells and the lack of increased Rho-activity in DLC1-GAP mutant HUVECs is the result of increased total Rho levels. The lack of an effect on Rho-GTP activity agrees with the thrombin data presented in fig 5 and previous data from the same lab showing no effect in Rho-activity upon DLC1 knockdown. Therefore, the graph 3e is misleading and should be removed together with the related statements in the results and discussion.
The above statements were supported by our data and we respectfully disagree with the reviewer on this matter. We have deliberately been showing the full analyses of RhoA-GTP levels, its total levels and their ratio as we observe consistent effects on overall Rho levels upon DLC1 overexpression. This is indeed unexpected, but nevertheless we did find that DLC1 expression, but not the GAP dead R677E mutant, leads to an increase in Rho-GTP and total RhoA levels (hence not their ratio). We don't have a full explanation for these findings, but we suspect that a compensation of the cells to the constitutive high DLC1 expression levels and GAP activity is at play. Others have previously observed similar compensatory effects of Rho

© 2024. Published by The Company of Biologists under the terms of the Creative Commons Attribution License (https://creativecommons.org/licenses/by/4.0/).

total protein upregulation as a result of the depletion of other Rho isoforms (Pronk et al., 2019). **We kept the experimental results and its discussion within the manuscript as it will be informative for the field. In analogue, others found a surprisingly faster Rho inactivation upon DLC1 depletion, and propose a GAP-mediated compensatory mechanisms as explanation (Heydasch et al 2023). To prevent possible confusion for readers, we now show the results of total RhoA levels instead of RhoA-GTP levels (Figure 5 d). Given the clear consensus that GEFs and GAPs act as spatiotemporal regulators, we chose to further delve into the aspect of Rho activity regulation using a location-based biosensor. As indicated in our earlier response above, these new experiments demonstrate that DLC1, but not the GAP dead R677E mutant, prevents induced Rho activation in the basal plane of the endothelial cells (Figure 5e, f).**

6. To determine whether there is an increase in P-MLC in DLC1-GAP mutant cells, given the increased total MLC levels, the ratio of P-MLC/MLC should be calculated. The increased of pMLC immunofluorescent could be a secondary effect of stabilised FAs and not a primary effect of DLC1-GAP activity.
We agree with the reviewer that the difference in pMLC2 intensity might be due to a secondary effect. Similarly, we observe a decrease in total MLC2 in DLC1 conditions on Western blot, but no effects on MLC2 phosphorylation levels. We have changed this results section, which now reads as follows:

"We did not observe downregulation of MLC2 phosphorylation in DLC1, or DLC1-R677E overexpressing cells (Sup. Fig. 4a-c), suggesting that DLC1 does not directly control MLC2 activation. Taken together, these findings point to an inhibitory role of DLC1's GAP function on Rho-ROCK-mediated phosphorylation of Cofilin."

We calculated the ratio of P-MLC2/MLC2, which is now presented in Supplemental Figure 4c.

7. In legend of figure 4 there is the statement: "Cells without any measureable tractions were not included in this quantification." However, in graph 4j, there are many zero values on DLC1 and DLC1-GAP mutant cells. The data in the graph should be corrected. Furthermore, the colour scale of traction forces should be corrected to depict better the data. As it is now, it shows that cells with zero traction forces are spread.
The included values the reviewer refers to are in fact not zero, but slightly higher, and thus the quantification does not need a correction. We have included the graph with a log-scale transformed y- axis to depict this (see Figure 1 for reviewer).

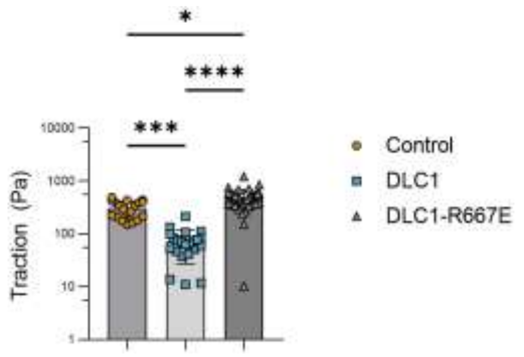

Figure 1 for Reviewers

In the original manuscript the color scale of traction forces was set from 0 to the maximum level of tractions detected in all conditions. To visualize the traction differences per cell type we have now included adjusted heatmap scales tailored to the representative images. Please note that the heatmaps show single tractions and the bar graphs depict the quantified average traction per cell (Figure 5i).
8. The authors should discuss the differences between the overexpression of the dominant negative DLC1- GAP mutant and the DLC1 knockdown published before (van der Stoel) on focal adhesion assembly/disassembly rates and the lifetime of focal adhesions.
Both in DLC1-depleted and in d.n. DLC1-R677E expressing endothelial cells we observed

© 2024. Published by The Company of Biologists under the terms of the Creative Commons Attribution License (https://creativecommons.org/licenses/by/4.0/).

reduced FA disassembly and increased focal adhesion lifetime compared to control conditions. Of note, we observed many long-lived stationary focal adhesions in the cell body of DLC1-R677E overexpressing cells, that were not detected by FAAS because they even did not turnover within the timeframe of the recordings. Nevertheless, the lifetime of the remaining focal adhesions was also slightly increased by DLC1-R677E (Fig. 4g; see the stronger difference of FA lifetime in DLC1 vs. DLC1-R667E expressing cells compared to DLC1 vs. control). There is indeed a differential effect on focal adhesion assembly in DLC1- depleted and d.n. DLC1-R677E expressing endothelial cells. Potentially, the dominant negative effect of DLC1-R667E expression, through its recruitment to focal adhesions, affects their assembly more profoundly than in the absence of DLC1, a point which we now addressed in the discussion section.

9. The authors should discuss how the reduced F-actin formation and FA stabilisation leading to reduced YAP translocation increase in vitro endothelial sprouting.
The experiments indicate that DLC1-driven feedback towards Rho (and YAP) promotes angiogenic sprouting. Likewise, experiments from other labs in which inhibition of Rho signaling by Y-27632 was performed, also increases sprouting in 3D (Guo et al., 2022). The increase in migration capacity can be attributed to the formation of short-lived focal adhesions (hence more turnover). The formation of very static and strong focal adhesions (such as in DLC1-R677E and DLC1-depleted cells) negatively affects cell migration and sprouting. This is reminiscent of related 3D migratory processes, for instance during metastasis, when tumor cells switch to different modes of motility and higher velocity is marked by a reduction in focal adhesions and actin bundles (Chikina & Alexandrova, 2014). We addressed this point in the discussion section.

3. Additional minor points:
1. In the quantification of TIRF experiments, indicate how many FAs were analysed per cell in the different conditions.
The average number of recorded focal adhesion events per cell for assembly, disassembly and lifetime are now listed in the figure description.

2. In the mosaic experiments, the authors should quantify the number of spheroids having control cells vs DLC1 and DLC1-GAP mutant cells in the periphery at the onset of VEGF stimulation. Also, the length of each sprout from RFP control and GFP-DLC1, DLC1-GAP mosaic spheroids should be calculated instead of the cumulative sprout length. This quantification would indicate better the defect in VEGF-stimulated endothelial cell migration.
We have replaced the graph, which now depicts the average sprout length per spheroid, the corresponding conclusions remain similar. We did not quantify the peripheral occupancy of control vs. DLC1 and DLC1-R677E of the spheroids at t=0 as it is unclear what the biological meaning is of the reported organization.

Reviewer 2 Advance Summary and Potential Significance to Field:
This exciting study evaluates the role of the RhoA GAP, DLC1, in endothelial cell migration and angiogenesis, via feedback regulation of the transcriptional regulator YAP. This is an elegant and focused study that provides critical mechanistic insight into both the mediators of the feedback loop previously discovered by the team and provides new insights into DLC1 biochemistry, and the mechanobiology of focal adhesion regulation. The authors characterize the RhoGAP activity of DLC1 and its effects on YAP nuclear translocation and transcriptional activity, focal adhesion dynamics, Rho-ROCK signaling, and cytoskeletal tension.

This work advances our understanding of endothelial migration regulation and establishes DLC1 activity as a crucial negative feedback mediator, downstream of YAP, which acts to modulate YAP/TAZ activity to regulate sprouting angiogenesis. The results support their conclusions by demonstrating that signaling through DLC1's RhoGAP domain leads to a reduction in the nuclear localization of YAP and its transcriptional activity, which influences YAP-mediated cellular processes such as regulating focal adhesion turnover, modulating traction forces, and facilitating sprouting angiogenesis. An exciting advance, this paper will be of great interest to the broad readership of the JCS and will be valuable to readers with interests in cytoskeletal and focal adhesion regulation, mechanobiology, and vascular biology.

© 2024. Published by The Company of Biologists under the terms of the Creative Commons Attribution License (https://creativecommons.org/licenses/by/4.0/).

Enthusiasm for the contributions of the paper is moderated by a rather confusing presentation. Increasing the clarity of the paper for this broad audience, who may not be familiar with the group's prior papers on YAP/TAZ-DLC1 feedback, will increase both its reach and impact. We provide some suggestions toward this end below, but emphasize the importance and quality of the paper and its relevance for the JCS community.

Reviewer 2 Comments for the Author:
This work builds the authors' previous discovery that Deleted-in-Liver-Cancer-1 (DLC1) is a direct target of the YAP mechanotransducer and promotes sprouting angiogenesis through a mechanotransductive feedback loop that regulates cytoskeletal contractility. The prior literature on this YAP-RhoA feedback loop, while mentioned in the introduction, are not sufficiently detailed for a general audience. Understanding these previous publications is key to understanding the starting point and context of the current work. The authors should make it easier for the reader to make this connection by adding a summary of the previous results in the introduction - because this is a feedback loop, the reader can become easily confused without clear description of where to start. We also suggest including a schematic as Figure 1 to illustrate what is currently known about the feedback loop and to identify the knowledge gap that will be addressed in this work.
We agree that we needed to expand on the relevant prior findings of the related literature in the introduction to better explain the connection to the current findings. We have adjusted the introduction to more clearly explain the concept of YAP/TAZ mechanotransductive feedback loops and the results from our earlier work with DLC1-depleted endothelial cells. We hope these changes sufficiently improved the context of the current study.
The authors aimed “to investigate the role of DLC1 in the modulation of YAP activity in endothelial cells during angiogenic sprouting”. The way the manuscript is currently structured makes it difficult to follow. We recommend a re-organization of the paper into two parts. Part 1 would focus on the role of DLC1 in the modulation of YAP activity [Figures 3, 1], establishing the GAP activity of DLC1 as a mediator of the YAP-RhoA feedback loop. Part 2 would then address the key gap of the paper, namely how does the RhoGAP activity of DLC1 function in regulation of cell contractility, tension generation, focal adhesion turnover, and functional sprouting [Figures 2, 4, 5].
We thank the reviewer for providing this advice. We have reorganized the order of the figures as follows: Figures 1-3 (role of DLC1’s RhoGAP function in feedback towards YAP activation and YAP- mediated endothelial sprouting); then the second part of the manuscript is addressing the underlying mechanism of DLC1-RhoGAP mediated regulation of Rho signaling, intracellular tension and focal adhesions.

The results from RhoA and RhoA-GTP quantification in HUVECs were confusing. For the aim of this paper, it would be unnecessary to do more experiments to understand these observations; however, the authors should make it clear why these results are unexpected and what they might mean. This is especially important because it is the basis for understanding the thrombin stimulation experiment (Figure 5b, c). Because this confusing result is the basis for the following argument: “DLC1 does not regulate global Rho signaling, but may control local intracellular contractility at focal adhesions,” it limits confidence in this conclusion. While it is clear from the data that DLC1 GAP activity regulates focal adhesions, the data do not directly show local DLC1 or RhoA activity IN or ON the focal adhesions. Because it is hard to interpret the global RhoA-GTP blot data, it is hard for us to follow the argument from the thrombin treatment experiment. This is further complicated because Thrombin is not a direct RhoA-agonist, but has a variety of signaling consequences in endothelial cells downstream of PAR-1).
This remark overlaps with a comment from reviewer 1. We apologize for not explaining the rationale for the thrombin experiment sufficiently clear. The purpose of the concluding experiments in Figure 6 were to investigate whether we could still bypass the DLC1-driven negative feedback pathway by inducing overall Rho-mediated contractility, thereby establishing whether this feedback loop is still subject to regulation. Thrombin is a well-known, but indeed not necessarily specific, activator of RhoA and actomyosin-mediated contractility in endothelial cells (Amerongen et al, 2000, Botros et al, 2020, Huveneers et al, JCB, 2012). Our current experiments confirm that thrombin-treatment activates overall RhoA-GTP loading (Supplemental Figure 2) and demonstrate that this restores nuclear YAP levels in DLC1 overexpressing sparse cells (Fig. 6c, d).

We decided to strengthen the evidence for a role of DLC1 on Rho signaling by performing

© 2024. Published by The Company of Biologists under the terms of the Creative Commons Attribution License (https://creativecommons.org/licenses/by/4.0/).

additional experiments. By using a location-based biosensor we now demonstrate that DLC1, but not the GAP dead R677E mutant, prevents induced Rho activation in the basal plane of the endothelial cell. The biochemical rhotekin pulldowns on cell lysates showed that thrombin promotes RhoA GTP-loading in control, DLC1 or DLC1-R677E conditions (Supplemental Figure 2). Together, the data thus indicate that DLC1 does not inhibit global Rho signaling, but controls the local activation of Rho GTPases at the basal plasma membrane. However, we did not find specific inhibition of Rho GTPases at the focal adhesions compared to other basal membrane regions, so we removed any such suggestions from the revised manuscript.

The concluding remarks focus on the role of DLC1 in cancer. The authors have not previously mentioned cancer in the manuscript and it takes focus away from the aim of the paper. We suggest rewriting or removing this section.
Previous work from others indicate that DLC1-defiency is associated with increased nuclear YAP in angiosarcoma (Ritchey et al 2019). We wanted to extend the possible relevance of our findings for targeting of the feedback pathway for this cancer type. To keep the discussion within the scope of the paper, we deleted the section mentioning of the role of DLC1 in solid tumors.

Discuss the potential roles of TAZ vs. YAP.
YAP and TAZ have distinct, but also overlapping transcriptional programs and depending on the context can even play contrasting roles. In this study we focus on how YAP activity is regulated by DLC1 through cell shape and mechanical regulation. While there are a few paralog-specific regulatory mechanisms, cell shape and mechanical state of the cell is not one of these. Also DLC1 expression levels depend on the activity of both YAP and TAZ (van der Stoel et al, 2020), thus we suspect DLC1 might also provide feedback to TAZ. We now eluded on this possibility in the discussion.

The authors “observed many long-lived stationary focal adhesions in the cell body that were not detected by FAAS”. Expand on the limitations of measuring focal adhesions using FAAS and how that impacts your results. Is there a way to measure these long-lasting FA's?
Indeed, the maximal lifetime parameter in FAAS is capped by the duration of the time lapse recordings, leading to an underestimation of the average lifetime in DLC1-R667E expressing cells. We added the following text to explain: “Furthermore DLC1-R677E induced many long-lived stationary focal adhesions in the cell body (Fig. 4e). Their lifetime could not be assessed as they even did not turnover within the timeframe of the recordings”. An alternative experimental setup, by recording longer movies, while reducing sampling time to prevent phototoxicity, might be able to solve this, but then the accuracy on FA turnover (determining (dis-)assembly rates) parameters will be less. Even though this is a limitation of the quantification approach, the quantified data support the observed finding that DLC1 RhoGAP activity promotes focal adhesion turnover and limits focal adhesion maturation. Nevertheless, the focal adhesion lifetime of the remaining focal adhesions also slightly higher in DLC1-R677E compared to DLC1 expressing cells.

Thrombin is not a specific RhoA activator. How does this impact your results and interpretation? Explanation of why thrombin was selected and what results were expected could help your readers follow the reasoning.
Please also see our earlier responses on this topic: Thrombin is a well-known, but indeed not necessarily specific, activator of RhoA and actomyosin-mediated contractility in endothelial cells (Amerongen et al, 2000, Botros et al, 2020, Huveneers et al, JCB, 2012). Our current experiments demonstrate that thrombin-treatment activates Rho-GTP loading and acts very quickly, as further evidenced by the new Rho biosensor experiments (which also showed clear cellular contractions upon thrombin-induced Rho activation. Alternative biologically relevant GTPase regulators, such as histamine or VEGF, also act via their corresponding receptors and act indirectly on GTP-loading.

Reviewer 3 Advance Summary and Potential Significance to Field:
The report by Hooglugt and colleagues describes the role of DLC1 in the regulation of YAP nuclear localization, in a process mediated by a RhoA/ROCK signaling pathway. The authors describe a negative feedback loop, by which DLC1 inhibits YAP’s nuclear localization, in particular during cell migration and angiogenesis. The process requires the GAP activity and it is proposed to work

© 2024. Published by The Company of Biologists under the terms of the Creative Commons Attribution License (https://creativecommons.org/licenses/by/4.0/).

through RhoA/ROCK. The study is significant as it describes a novel role for DLC1/YAP and may explain tight regulation required that is required to control angiogenesis.

Reviewer 3 Comments for the Author:

General remarks

The experiments are well designed for the most part and the results are solid in general. However, there are two important aspects of this study that, in my opinion, need to be revised to further support the conclusions made by the authors: First, most experiments are based on results from overexpression of a RhoGAP. The question is whether by grossly overexpression a RhoGAP, the effects observed are due to a global, non-specific inactivation of RhoA in the cell. Second, there is uncertainty, and also lack of clarity, regarding whether DLC1 modulates RhoA activity locally, or another RhoGTPase in HUVECS, as no inhibitions of RhoA is observed in DLC1 overexpressing cells. Addressing these concerns will provide a clearer picture of the pathway involved and will strengthen the manuscript significantly.

We thank the reviewer for these constructive questions. To address them we performed a series of new experiments, which gave clear answers on these points, see our answers to the corresponding specific comments:

Specific comments:

The role of RhoA downstream DLC1 is the weakest aspect of this study. The experiments showing that DLC1 overexpression does not inactivate RhoA in HUVECs are confusing. It is hard to understand how high total RhoA-GTP in DLC1 overexpressing cells correlates with less actin polymerization. The authors also state that RhoB may be affected but the differences in expression levels are not significantly different and the activity was not tested. It may be possible that the activity of RhoB and RhoC are decreased and that could explain the effects on YAP activation. My recommendation is to perform additional experiments to confirm or rule out the direct role of RhoA or other related GTPases in the regulations of the events downstream of DLC1. 1) Measure the local inactivation of RhoA using a RhoA sensor. I still believe that the most likely possibility is global inactivation of RhoA/B or C based on the high levels of overexpression, but the authors propose local inactivation of RhoA by DLC1. A Rho sensor should be able to distinguish between these scenarios. 2) Measure the activation levels of RhoB and C. RhoC appears to be relevant as it has been shown to regulate YAP through the function of another GAP ARHGAP18 (https://doi.org/10.1186/s12964-020-0511-7).

1) As mentioned before in our answers to reviewers 1-2, we strengthened the evidence for the role of DLC1 on Rho signaling by performing additional experiments. By using a location-based biosensor we now demonstrate that DLC1, but not the GAP dead R677E mutant, prevents induced Rho activation in the basal plane of the endothelial cell. The biochemical rhotekin pulldowns on cell lysates showed that thrombin promotes RhoA GTP-loading in control, DLC1 or DLC1-R677E conditions (Supplemental Figure 2). Together, the data thus indicate that DLC1 does not inhibit global Rho signaling, but controls the local activation of Rho GTPases at the basal plasma membrane.
2) **In other cell types DLC1 has been shown to inhibit GTP-loading of the RhoB and RhoC-GTP isoforms (Amin et al., 2016; Healy et al., 2008; Wong et al., 2003). From previous experiments, in which we knocked down the expression of endogenous DLC1, we learned that DLC1-depletion does not regulate global RhoB or RhoC GTP-loading in HUVECs either (data not shown). To address the specific comment of the reviewer we performed additional biochemical RhoB and RhoC pull downs from control, DLC1 and DLC1-R667E overexpressing HUVECs. However, we observed no reduction in GTP loading for RhoB or RhoC upon DLC1 overexpression (see representative Figure 2 for reviewer). As most DLC1 literature has focused on its GAP function towards RhoA, we now focus on RhoA as primary readout for the pull downs, and removed data concerning the other Rho isoforms, to improve the clarity of the manuscript. Of note, the newly included Rho biosensor is based on rhotekin, and thus reports on the activity of all three RhoA/B/C isoforms. Hence we conclude on Rho signaling instead of mentioning the specific isoforms here.**

© 2024. Published by The Company of Biologists under the terms of the Creative Commons Attribution License (https://creativecommons.org/licenses/by/4.0/).

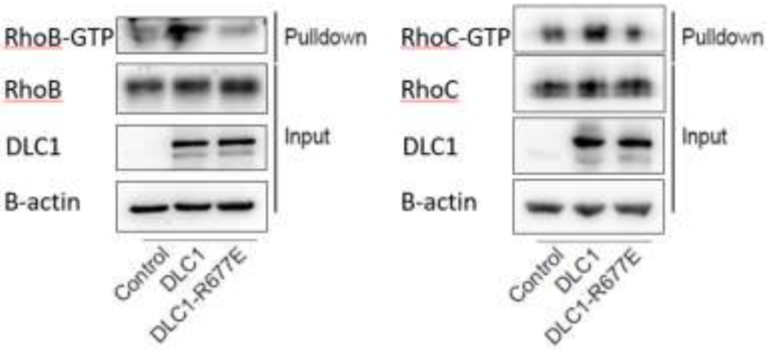

Figure 2 for Reviewers

A second key concern is the level of overexpression of DLC1 and DLC1 mutants. In particular since these are not stably expressing cell lines, the levels of overexpression (or rescue) detected by WB correspond only to a fraction of the cells, so they may be even higher on a per cell basis. For example, in Supp. Fig. 1h there are probably several fold overexpression levels in the KD/rescue experiment. At these levels of overexpression, the question is whether the effects observed are specific to DLC1 function, or to a non- specific global inhibition of RhoA activity in cells. This can be tested by overexpressing a non-related RhoA GAP (or just the GAP domain of DLC1). If the effects are non-specific for DLC1 then the non-specific GAP should not phenocopy DLC1 overexpression.

Based on the notion that overexpression of DLC1 has the opposite effect on F-actin and focal adhesions as compared to knocking down endogenous DLC1 (van der Stoel, JCS 2020), we can conclude that the observation are specific for DLC1. In addition, the overexpression of d.n. DLC1-R677E recapitulates DLC1 knockdown cells. Furthermore, the protein conformation of DLC1 is auto-inhibited and its GAP domain can only turn catalytically active when the protein is phosphorylated by CDK5, which concurrently causes focal adhesion localization (Tripathi et al JCB 2014). This means that activation of the overexpressed DLC1 is still subject to regulatory signaling pathways. Also the notion that we don't observe global suppression of RhoA activity upon DLC1 overexpression in the pull downs, but we did observe suppression of Rho at the basal cellular plane where active DLC1 localizes, strengthens the notion that the reported effects are specific for DLC1.

In addition, to address this point experimentally, we generated new lentiviral plasmids expressing only the GAP domain of wild type or catalytically inactive DLC1. Upon their expression, we observed that these GAP domain variants are cytoplasmic and did not localize at the focal adhesions (Supplemental Figure 3a, b). Moreover, both GAP variants were enriched in the nucleus, whereas the full length DLC1 protein counterparts did not, indicating that the other regions within the DLC1 protein contribute to its subcellular localization. Expression of the GAP domain of DLC1 reduced F-actin and the formation focal adhesions (Supplemental Figure 3b, c). Moreover, the GAP domain strongly induced membrane ruffles containing small punctate focal contacts at the cell periphery (Supplemental Figure 3c), indicating that expression of only the GAP domain had even stronger effects than full length DLC1, possibly due to the absence of its auto-inhibitory domains. In addition, the GAP domain inhibits YAP-mediated CTGF expression (Supplemental Figure 3d, e) and basal Rho activation by thrombin (Movie 3; Supplemental Figure 3f, g). Conversely, expression of the catalytic inactive GAP-R677E domain promoted focal adhesion formation, stress fibers and did not inhibit thrombin-induced basal Rho activation. These results show that the presence of DLC1 GAP activity in the cytoplasm is sufficient for the reported endothelial feedback responses.

One concern in general regarding the overexpression experiments is that he levels of transfection efficiency (at least in the pictures shown) seems to vary a lot. This is not a problem for immunofluorescence, but it may represent a problem in the biochemical assays, because depending on the efficiency of transfection, the effects may be more or less pronounced (see for example the difference of expression levels between DLC1 and 677 in Fig. 1i. This could represent differences of transfection efficiency, expression/stability differences between WT and 677, or a combination of both.

The images of immunofluorescent stainings show the result of lentiviral transductions, which

© 2024. Published by The Company of Biologists under the terms of the Creative Commons Attribution License (https://creativecommons.org/licenses/by/4.0/).

indeed results in varying levels of overexpression of DLC1 per cell. Of note the distinctive morphological changes upon the expression of DLC1 (resolution of F-actin, focal adhesions and less adhesive) vs. DLC1- R677E (increased stress fibers, focal adhesions and more spread) was consistent for different expression levels. For the biochemical assays, the transduced HUVECs have been selected with puromycin to guarantee that all cells ectopically express GFP-DLC1 or DLC1R677E-GFP. As these are not stable lines but primary cells, we further controlled the overexpression levels by adjusting the virus titer. After puromycin selection, we verified whether the monolayers were GFP positive and adopted the characteristic DLC1 or DLC1-R677E overexpression morphology prior to performing biochemical experimentation. While expression levels sometimes still varied a bit between experiments, we made sure that it did not affect the experimental conclusions. For instance, even though overexpression of DLC1 in Fig1e was relatively low compared to DLC1-R677E, it still strongly downregulated CTGF expression, corresponding with its negative feedback towards YAP activity.

There are complementary experiments silencing the expression of DLC1, which I believe are more physiologically relevant than the overexpression ones. However, in this manuscript, they are all presented as Supplemental Figures and the emphasis is placed on the overexpression experiments. I believe that some, or all of them should be in the main figures.

We thank the reviewer for the suggestion. We have now moved the rescue experiments to the main figures. We initially chose to only present the results from the overexpression experiments as major figures, instead of the elegant rescue (shRNA of endogenous DLC1 and rescue with lentviral expression) experiments, because we observed similar effects for DLC1 expression on YAP localization, sprouting angiogenesis and cytoskeletal morphology under both conditions. Of note, we are constraint by the fact that generating rescues 'lines' from these primary cells is very laborious, which limits their availability for more complicated experiments such as the Rho activation assays or live imaging for which a larger quantity of cells are needed or subsequent genetic modifications.

It is hard to judge a result like the one in Supp. Fig. 2a without quantification and replication. Some panels show just one cell. Maybe FA number and/or size could be quantified as done in Fig 2. Similarly, quantification of spreading and focal adhesion dynamic changes (or lack thereof) should be provided to support the conclusion in Fig. 5a and Supp. 5a.

We understand that we can quantify more aspects of these cells, but also want to keep the focus on the experiments on which we have based the key conclusions. Therefore, we now added quantifications of the cumulative focal adhesion area in the time lapse recordings (at t=0 and t=30 min) following thrombin treatments in DLC1 and DLC1-R677E expressing cells (Figure 6b). The quantifications support the claim that upon addition of thrombin to DLC1 overexpressing cells, an increase in focal adhesion number and size was observed.

The data in Supplemental Figure 1 were meant to illustrate the cellular phenotypes of the DLC1 knockdown and rescued cell types. All the corresponding functional data have been quantified (sprouting and YAP translocation). Please note that the images of Supplemental Figure 1 are representatives of at least 3 independent experiments.

Indeed we have not quantified cell spreading in Fig5 and S5. Hence, we have removed conclusions from the text that were referring to cell spreading. The Y-27632 inhibitor experiments confirmed for us that the FAs in DLC1-R677E cells depend on Rho-ROCK activity, whereas there is no such response in DLC1 (as they already have little Rho-Rock activity and focal adhesions). We have removed these experiments from the revised manuscript as they are not needed to support its claims.

Second decision letter

MS ID#: JOCES/2023/261687

MS TITLE: DLC1 promotes mechanotransductive feedback for YAP via RhoGAP-mediated focal adhesion turnover

© 2024. Published by The Company of Biologists under the terms of the Creative Commons Attribution License (https://creativecommons.org/licenses/by/4.0/).

AUTHORS: Aukie Hooglugt, Miesje M. van der Stoel, Apeksha Shapeti, Beau Neep, Annett de Haan, Hans van Oosterwyck, Reinier A Boon, and Stephan Huveneers

I am happy to tell you that your manuscript has been accepted for publication in Journal of Cell Science, pending standard ethics checks.

Reviewer 1

Advance summary and potential significance to field

The authors have addressed all my comments satisfactorily and have performed a new series of experiments that clarify all the issues raised. The manuscript text is now more clear and concise. I endorse this paper for publication.

Comments for the author

The authors have performed critical experiments that have addressed all my comments. Without any hesitation I endorse this manuscript for publication. I believe that it would be a great addition of knowledge to the field.

Reviewer 2

Advance summary and potential significance to field

The significance has been clarified and amplified by this revision.

Comments for the author

The authors have satisfied all my concerns. The authors are to be congratulated on an exciting study and significant contribution to our understanding of cytoskeletal regulation.

Reviewer 3

Advance summary and potential significance to field

The report by Hooglugt and colleagues describes the role of DLC1 in the regulation of YAP nuclear localization, in a process mediated by a RhoA/ROCK signaling pathway. The authors describe a negative feedback loop, by which DLC1 inhibits YAP's nuclear localization, in particular during cell adhesion, migration and angiogenesis. The process requires the GAP activity and it is proposed to work through RhoA/ROCK.
The study is significant as it describes a novel role for DLC1/YAP and may explain tight regulation required that is required to control angiogenesis.

Comments for the author

In this revised version, the authors have systematically addressed all reviewers concerns either in writing or through additional experiments. I believe this version is significantly improved and the manuscript should be ready for publication.
There is one exception though. In the revision the authors added experiments that use a Rho localization sensor to demonstrate the GAP activity inhibits the activity of Rho locally. By comparing the signal of the sensor at the ventral side of the cell using TIRF (inhibited by DLC1 OX), with the total activity of RhoA using a pulldown assay (not major change with DLC1 OX), they conclude that DLC1 only regulates the activity of RhoA at the basal adhesion plane.

© 2024. Published by The Company of Biologists under the terms of the Creative Commons Attribution License (https://creativecommons.org/licenses/by/4.0/).

I think that this comparison of imaging vs. biochemistry is not sufficient to support this conclusion. The results of the biosensor are striking, and in my opinion do not rule out that RhoA is activated in the whole cell. A better control would have been to compare the signal of the sensor using epifluorescnece (whole cell) vs TIRF (basal). The pulldown assay is not as sensitive as the biosensor and I don't think a suitable comparison.
Adding the experiment would be ideal to support their conclusions, but this can also be addressed in writing. However, this would require to tone down theirs conclusions and address the limitations of their experimental approach and interpretation.

© 2024. Published by The Company of Biologists under the terms of the Creative Commons Attribution License (https://creativecommons.org/licenses/by/4.0/).
